# Egg cell-specific promoter-controlled CRISPR/Cas9 efficiently generates homozygous mutants for multiple target genes in *Arabidopsis* in a single generation

**DOI:** 10.1186/s13059-015-0715-0

**Published:** 2015-07-21

**Authors:** Zhi-Ping Wang, Hui-Li Xing, Li Dong, Hai-Yan Zhang, Chun-Yan Han, Xue-Chen Wang, Qi-Jun Chen

**Affiliations:** State Key Laboratory of Plant Physiology and Biochemistry, College of Biological Sciences, China Agricultural University, Beijing, 100193 China

## Abstract

*Arabidopsis* mutants produced by constitutive overexpression of the CRISPR/Cas9 genome editing system are usually mosaics in the T1 generation. In this study, we used egg cell-specific promoters to drive the expression of *Cas9* and obtained non-mosaic T1 mutants for multiple target genes with high efficiency. Comparisons of 12 combinations of eight promoters and two terminators found that the efficiency of the egg cell-specific promoter-controlled CRISPR/Cas9 system depended on the presence of a suitable terminator, and the composite promoter generated by fusing two egg cell-specific promoters resulted in much higher efficiency of mutation in the T1 generation compared with the single promoters.

**Electronic supplementary material:**

The online version of this article (doi:10.1186/s13059-015-0715-0) contains supplementary material, which is available to authorized users.

## Background

The large collections of *Arabidopsis* sequence-indexed T-DNA insertion mutants (over 325,000 lines) have played a critical role in direct investigations of gene function [1]. However, two major obstacles limit the application of these collections for genome-wide phenomic screening. First, most lines are hemizygous for the insertion, and thus have required an extra genotyping step to identify homozygous plants for phenotyping. Second, no T-DNA insertion mutants are available for 12 % of genes, and 8 % of genes are only represented by a single allele [2]. Additionally, dissecting the roles of gene family members with redundant functions and analyzing epistatic relationships in genetic pathways frequently require plants bearing mutations in multiple genes. One hindrance to producing multi-gene mutants using T-DNA insertion mutagenesis is that this method requires time-consuming and labor-intensive genetic crossing of single-mutant plants. Advances in the use of sequence-specific nucleases, including homing meganucleases, zinc finger nucleases (ZFNs), transcription activator–like effector nucleases (TALENs), and, most recently, the RNA-guided DNA endonuclease Cas9 from the bacterial adaptive immune system CRISPR (clustered regularly interspaced short palindromic repeats) have paved the way for the development of rapid, cost-effective ways to create new mutant populations and multi-gene mutants in plants [3–7].

The CRISPR/Cas9 system uses an engineered single guide RNA (sgRNA) to provide sequence specificity, and depends on the endonuclease activity of the sgRNA/Cas9 complex to produce double-strand breaks at genomic sites specified by sgRNAs [7–10]; these double-strand breaks cause the activation of the DNA repair system in host cells, usually via the non-homologous end-joining pathway [5]. Since the repair pathway is error-prone, small deletions or insertions will be introduced during the repair process, thus producing mutations [5]. This highly efficient, easy-to-use system can potentially be used to make highly multiplexed genome modifications, and is supplanting the use of ZFNs and TALENs to become the standard genome-editing technology [3, 4, 6, 7]. In vertebrates, coinjection of *in vitro* transcribed *Cas9* mRNA and sgRNA into single-cell embryos can produce multi-gene, biallelic mutant animals with high efficiency; the multiple mutations can also be efficiently transmitted to the next generation [11–16]. In plants, however, the presence of the cell wall makes methods using RNA injection impractical. Creating transgenic lines expressing the CRISPR/Cas9 system provides an alternative method [17–44].

*Agrobacterium*-mediated techniques used to create transgenic plant lines include *in planta* transformation and embryogenic callus-based transformation. The most typical example of *in planta* transformation is *Agrobacterium*-mediated transformation of *Arabidopsis*, whose egg cell is the target of the T-DNA [45–48]. Embryogenic cell-derived transgenic lines expressing the CRISPR/Cas9 system can be homozygous for edited target genes in the first generation, indicating that the target genes were edited in the transformed embryogenic cells before the first cell division [28]. Similar results were reported in tomato [33] and maize [26]. These results are encouraging for the development of crop genome editing, since crop transformation usually uses embryogenic callus cells, which can be considered to be analogous to animal embryos at the one-cell stage. In *Arabidopsis*, which is highly amenable to *in planta* transformation, the CRISPR/Cas9 system should theoretically be able to function in one-cell stage embryos. However, transgenic lines expressing CRISPR/Cas9 have mainly been mosaic in the first generation (T1), indicating that CRISPR/Cas9-induced mutations in *Arabidopsis* occurred after the first embryonic cell division [20, 22, 25, 26, 29, 35]. Perhaps the failure of CRISPR/Cas9 to function in one-cell stage embryos was due to the weak activity of the constitutive Cauliflower Mosaic Virus 35S promoter (CaMV 35S) in egg cells and one-cell stage embryos.

In this study, we used the promoter of the egg cell-specific *EC1.2* gene [49, 50] to drive the expression of *Cas9* in *Arabidopsis*, demonstrating that the specific expression of CRISPR/Cas9 in egg cells and one-cell stage embryos could efficiently lead to the creation of homozygous or biallelic mutants for multiple target genes in *Arabidopsis* in the T1 generation. Identification of completely mutated, non-mosaic lines will usually require medium-depth sequencing of target loci in a few candidate lines screened from 25**–**50 T1 transgenic plants via restriction enzyme digestion analysis, T7E1 assay, or Surveyor assay. However, the present strategy could shorten the time required to produce such mutants to a single generation, thus providing a quicker, more cost-effective means of creating new mutant populations and multi-gene mutants in *Arabidopsis*. Based on comparisons of different combinations of promoters and terminators, we also present a route to optimize the egg cell-specific promoter-controlled (EPC) CRISPR/Cas9 system.

## Results

### Targeted mutations of multiple *Arabidopsis* genes in the T1 generation

Two reports have demonstrated that *DD45*/*EC1.2* (At2g21740) is an egg cell-specific gene [49, 50]. *In situ* hybridization of tissue sections revealed that *EC1.2* transcripts are specifically present in the egg cell, whereas GUS activity and GFP signals were observed in EC1.2p:GUS and EC1.2p:GFP transgenic zygotes and early embryos; the carryover of the signal into later stages of embryogenesis likely resulted from higher stability of the reporter mRNA and/or protein [49, 50]. We reasoned that *Cas9* driven by the *EC1.2* promoter would be specifically transcribed in the egg cell; *Cas9* mRNA would reside in one-cell stage embryos due to mRNA stability and continue to translate Cas9 protein. Also, newly translated Cas9, together with residual Cas9 that remained due to Cas9 protein stability, would function in one-cell stage embryos, thus allowing creation of *Arabidopsis* T1 homozygous or biallelic mutants, rather than mosaic plants.

**Fig. 1:**
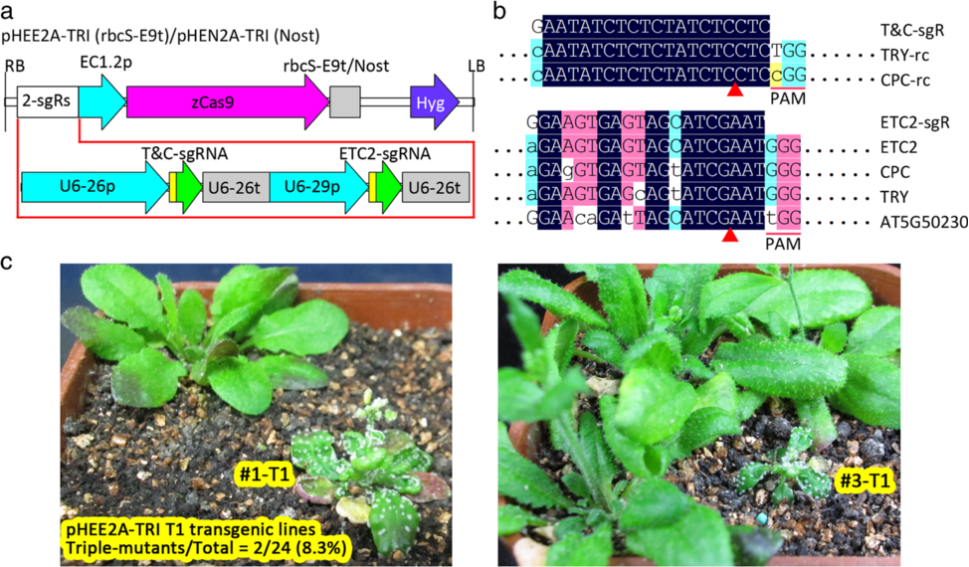
*Arabidopsis* T1 homozygous triple mutants obtained via EPC CRISPR/Cas9. **a** Physical maps of the T-DNAs of two CRISPR/Cas9 binary vectors, each harboring *Cas9* driven by the egg-cell specific promoter EC1.2p and two sgRNA genes driven by Pol-III promoters U6-26p and U6-29p, respectively. RB/LB, T-DNA right/left border; EC1.2p, *EC1.2* promoter; rbcS-E9t, *rbcS E9* terminator; Nost, *nos* gene terminator; sgR, sgRNA; 2-sgRs, two sgRNA expression cassettes; zCas9, *Zea mays* codon-optimized *Cas9*; U6-26p and U6-29p, two *Arabidopsis U6* gene promoter; U6-26t, *U6-26* terminator with downstream sequence; *Hyg*, hygromycin-resistance gene. For the sgRNAs, the yellow part represents 20-bp target and the green part represents 76-bp sgRNA scaffold. **b** The alignment of the sgRNA with its target genes and potential off-targets. Only aligned regions of interest are displayed. rc, reverse complement. **c** Phenotypes of two triple mutants segregated from T1 transgenic lines. The other plants in the same pot are from the same batch of T1 transgenic lines with normal phenotypes. Seeds from the T0 plants were sown on MS medium containing 25 mg/L hygromycin, vernalized at 4 °C for 3 days, and grown under long-day conditions (16 h light/8 h dark) at 22 °C for 9 days. Hygromycin-resistant seedlings (T1) were transplanted to soil and allowed to grow for 33 days before photographing

Since combinations of the same promoter with different terminators might result in significantly different amounts of protein [51], we made the two constructs to examine the effects of two terminators, the *Pisum sativum rbcS E9* terminator, in the pHEE2A-TRI construct, and the *Agrobacterium nos* gene terminator, in the pHEN2A-TRI construct, on the expression of *Cas9* driven by the *EC1.2* promoter (Fig. 1). We used two single guide RNAs (sgRNAs) to target three genes, *ETC2*, *TRY*, and *CPC* (Fig. 1a, b), since the *try cpc* double and *etc2 try cpc* triple mutants have easily observed phenotypes (clustered leaf trichomes) and the triple mutant has a different phenotype from the double mutant [26].

In our first attempt, we obtained 24 T1 EC1.2p:zCas9-rbcS_E9t lines using pHEE2A-TRI (Fig. 1a) and 54 T1 EC1.2p:zCas9-Nost lines using pHEN2A-TRI (Fig. 1a). Among the 24 T1 EC1.2p:zCas9-rbcS_E9t lines, two lines (#1 and #3) were likely triple mutants (Fig. 1c), and one line (#21) was a mosaic with two branches that displayed the double-mutant and wild-type phenotypes, respectively. We sequenced the regions surrounding the target sites of the three genes from the two putative triple mutant lines, and confirmed that they were indeed triple mutants (Table 1). In this instance, all observed mutations were single base pair insertions or deletions. Sequencing analysis and examination of the phenotypes of T2 plants derived from these two T1 lines further confirmed the identity of the two mutant lines (Tables 1 and 2). Unexpectedly, we failed to identify a likely triple mutant, double mutant, or mosaic among 54 T1 EC1.2p:zCas9-Nost lines, suggesting that the combination of the *EC1.2* promoter and *rbcS E9* terminator performed much better than the combination of the *EC1.2* promoter and *nos* terminator.

**Table 1:** Mutation analysis of three T1 likely triple mutants and their T2 progeny

Line		*ETC2*	*TRY*	*CPC*	Genotype
T1	#1	+A/+C	−C/−C	+A/+T	eettcc
T2	1-1	+A/+C	−C/−C	+A/+T	eettcc
	1-2	+A/+A	−C/−C	+A/+A	eettcc
	1-3	+A/+C	−C/−C	+A/+T	eettcc
	1-4	+A/+A	−C/−C	+A/+A	eettcc
T1	#3	+T/+T	+T/+G	+G/+T	eettcc
T2	3-1	+T/+T	+G/+G	+G/+T	eettcc
	3-2	+T/+T	+G/+G	+T/+T	eettcc
	3-3	+T/+T	+G/+G	+G/+T	eettcc
	3-4	+T/+T	+G/+G	+G/+T	eettcc
T1	#C1	+C/+A	+T (×13)/−G (×11)	+G/+G	eettcc
T2	C1-17	+A/+A	+T/+T	+G/+G	eettcc

All mutations but *TRY* from #C1 in this experiment were single-base insertions or deletions by direct sequencing of PCR products and the inserted (+) or deleted (−) nucleotide is denoted. *TRY* mutations in #C1 were detected by sequencing of cloned PCR products, and the number of the same type of mutation is indicated in parentheses. Two types of mutations from direct sequencing of PCR products were obtained based on double-peaks on chromatograph. Two alleles are separated by ‘/’. eettcc corresponds to *etc2 try cpc* triple mutant. C1-17 is a nontransgenic T2 line derived from #C1

**Table 2:** Phenotypic segregation analysis of T2 transgenic lines

T1	LTMs/Total-T2	T1	LTMs/Total-T2	T1	LTMs/Total-T2
1	20/20 (100 %)	9	0/83 (0)	17	11/34 (32.4 %)
2	0/162 (0)	10	33/156 (21.2 %)	18	0/42 (0)
3	20/20 (100 %)	11	27/49 (55.1 %)	19	0/53 (0)
4	42/98 (42.9 %)	12	0/78 (0)	20	0/36 (0)
5	15/47 (31.9 %)	13	0/52 (0)	21	18/45 (40.0 %)
6	25/77 (32.5 %)	14	0/202 (0)	22	n.a.
7	13/64 (20.3 %)	15	0/57 (0)	23	57/94 (60.6 %)
8	0/56 (0)	16	0/38 (0)	24	53/90 (58.9 %)

LTMs, likely triple mutants, that is, T2 plants with phenotypes similar to those of *try cpc etc2* triple mutants; Total-T2, total number of T2 plants examined; n.a., not available. The average segregation ratio of the LTMs to total T2 plants examined was 24.8 % ((100 % + 100 % + 42.9 % + … + 58.9 %)/24). The T2 seeds from #1 and #3 were sown on MS medium whereas those from the other T1 lines were sown on hygromycin (25 mg/L) medium

To examine the specificity of the mutagenesis, we searched the *Arabidopsis* genome for potential off-targets with fewer than four mismatches with the targets of the sgRNAs. This identified three potential off-targets of the sgRNA targeting *ETC2* [52]. We sequenced these regions in the two triple mutants and found no mutations, demonstrating the high specificity of the EPC CRISPR/Cas9 system.

To confirm the repeatability of the results from EC1.2p:zCas9-rbcS_E9t transgenic lines, we performed two additional, independent *Arabidopsis* transformation experiments with the construct pHEE2A-TRI. In the second transformation, we obtained 41 T1 lines, among which three were likely triple mutants (Additional file 1: Figure S1). In the third transformation, we obtained 43 T1 lines, including four that were likely triple mutants (Additional file 1: Figure S2). Therefore, approximately 8.3 % (9/108) of the T1 plants were likely homozygous triple mutants.

We also demonstrated the usefulness of the EPC CRISPR/Cas9 system by performing targeted mutation of two *Arabidopsis* genes, *CHLI1* and *CHLI2*, in T1 plants. Simultaneous disruption of *CHLI1* and *CHLI2* leads to an albino phenotype. We obtained 99 T1 lines, including 18 putative *chli1 chli2* double mutants (albino plants, see Additional file 1: Figure S3). We sequenced the regions surrounding the target sites, and found that 10 lines were double mutants and five were mosaic plants (Additional file 2: Table S1). These results indicate that the EPC CRISPR/Cas9 was functional, not only in one-cell stage embryos, but also in some early embryos, likely due to *Cas9* mRNA and/or protein stability, and/or reduced egg cell-specificity. Among the 18 albino lines, three grew poorly, and we were unable to obtain sequence data from these lines (Additional file 1: Figure S3, Additional file 2: Table S1). These three albinos were most likely double mutants rather than mosaics, based on their poor growth. Thus, the ratio of homozygous T1 double mutants to T1 plants was approximately 13 % (13/99). Together, these results demonstrate that our EPC CRISPR/Cas9 system could be used to efficiently produce confirmed T1 homozygous or biallelic mutants in less than 3 months (Additional file 3: Figure S4). In practical applications, users might have no visible phenotypes that they could use to screen for T1 homozygous mutants. However, this obstacle can be easily overcome by screening 25–50 T1 lines by restriction enzyme digestion analysis, T7E1 assay, or Surveyor assay (Additional file 3: Figure S5). After these primary screens, users will be able to quickly obtain a few candidate lines for sequence analysis (including direct sequencing of PCR fragments, sequencing individual clones of PCR fragments, and deep sequencing of PCR fragments) (Additional file 3: Figure S5).

To confirm that the T1 mutations are germline transmissible, we sowed 20 T2 seeds per T1 line derived from the two T1 triple mutant lines (#1 and #3) on MS plates. We observed no phenotypic segregation of these T2 plants (Table 2). Moreover, sequencing analysis of four T2 plants per T1 line showed no novel mutation types (Table 1). These results strongly suggested that germline transmission of T1 mutations occurred. To further confirm the germline transmission of the T1 mutations, we screened for non-transgenic T2 lines and analyzed their mutations. Since we harvested <30 T2 seeds per T1 line from the two triple mutant T1 lines (#1 and #3) due to their poor growth, no additional T2 seeds were available for screening of non-transgenic lines. We then turned to screening for non-transgenic T2 plants derived from the T1 triple mutant (#C1) produced in the third transformation (Additional file 1: Figure S2). We sowed 36 T2 seeds on MS plates, and transplanted the seedlings to soil. All 36 T2 plants were phenotypically triple mutants. We screened all 36 T2 plants for non-transgenic plants and obtained only one such plant, much fewer than the nine or so plants we expected, which may reflect insertions of two or more copies of T-DNAs into the genome of the T1 plant. We analyzed the mutations of the T1 mutant (#C1) and the non-transgenic T2 mutant (#C1-17) by sequencing (Table 1), which demonstrated that the T2 mutations are derived from the originally confirmed, rather than newly produced, T1 mutations through germline transmission (Table 1).

### Analysis of mutations in the phenotypically wild-type T1 plants and their T2 progeny

**Fig. 2:**
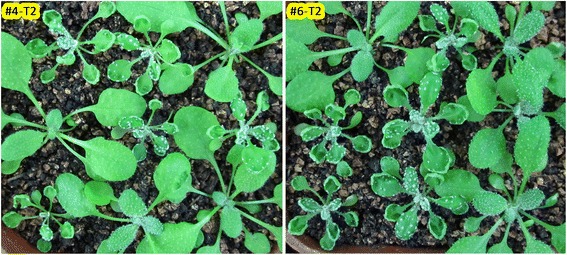
Phenotypic segregation of T2 transgenic lines. Phenotypic segregation of T2 transgenic lines derived from two representative T1 lines with normal phenotypes. Seeds from T0 plants were sown on MS medium containing 25 mg/L hygromycin, vernalized at 4 °C for 3 days, and grown under long-day conditions (16 h light/8 h dark) at 22 °C for 7 days. Hygromycin-resistant seedlings (T1) were transplanted to soil and allowed to grow for 20 days before photographing

Since CRISPR/Cas9 should continue to function in T1 egg cells, T2 one-cell stage embryos, and T2 early embryos, and since T1 plants with normal phenotypes might be heterozygotes or mosaics rather than wild type, T1 plants with no clear phenotypes should be able to give rise to homozygous or bi-allelic triple mutant T2 plants. To ensure that triple mutants could be differentiated from double mutants, we re-examined the phenotypes of the triple/double mutants, finding no differences from our previous observations (Additional file 3: Figure S6). Then, we examined T2 plants derived from the 24 T1 EC1.2p:zCas9-rbcS_E9t lines, revealing that approximately 50 % (12/24) produced likely triple mutant T2 progeny (Fig. 2 and Table 2). The segregation ratio of the likely triple mutants to total T2 plants examined was higher than 20 % for each of the 12 T1 lines and averaged 24.8 % for all 24 T1 lines (Table 2). Of the 54 T1 EC1.2p:zCas9-Nost lines (>100 T2 plants per line examined), only two lines, equivalent to 3.7 % (2/54), produced likely triple mutants in their T2 progeny. These results further demonstrate that the combination of *EC1.2* promoter and *rbcS E9* terminator performed much better than the *EC1.2* promoter and *nos* terminator combination, suggesting that in egg cells, the terminator is a key factor in stabilizing the *Cas9* mRNA and thus enhancing its translation.

We sequenced the three target genes of two representative T1 lines (#4 T1 and #6 T1), which had normal phenotypes (Table 3). We also sequenced their likely triple mutant T2 progeny (10 T2 plants per line; Table 3). The sequencing results revealed that the two T1 lines were mosaic with different degrees of mutation in the three target genes, demonstrating that the mutation frequency of a single gene in the T1 population was much higher than the frequency of simultaneous mutations of all three target genes. The formation of mosaic plants could be attributed to *Cas9* mRNA and protein stability (Additional file 2: Table S2). For example, for a two- or four-celled embryo derived from a zygote that had undergone two or three rounds of mitosis, each of the two or four cells would contain three-quarters or half the amount of Cas9 protein of that in the egg cell (if *Cas9* mRNA and protein were sufficiently stable; Additional file 2: Table S2). Two types of mosaic plants resulted from EC1.2p:Cas9 transformation: mosaics with a wild-type allele of a target gene and mosaics without wild-type alleles, which could be regarded as homozygous mutants. Analysis of the mutations present in the T2 progeny of the T1 mosaic plants demonstrated that most of the triple-mutant-like T2 plants were homozygous or biallelic triple mutants (Table 3).

**Table 3:** Mutation analysis of likely triple mutants segregated from two representative T1 lines with normal phenotypes

Line		*ETC2* mutation	*TRY* mutation	*CPC* mutation	Genotype
T1	#4	0/0	+G (×3)//+A (×9)//−G (×7)//+C (×2)	0/0	EEtxCC
T2	4-1	+C/+T	−G/−G	+A/+A	eettcc
	4-2	+A/+A	−G/−G	−8 (×8)/−G + T (×2)	eettcc
	4-3	+T (×9)/+5 (×11)	+A (×7)/−5 (×3)	+G (×4)/−14 + 4 (×6)	eettcc
	4-4	−41 (×9)/−24 (×11)	−G/−G	−G/−G	eettcc
	4-5	−14 + A (×13)/+A (×7)	−G/−G	−3/−3	eettcc
	4-6	−5/−5	+G (×8)/−G (×2)	+G/+A	eettcc
	4-7	+T/+A	+C/+C	+G/+T	eettcc
	4-8	+T/+T	−G/−G	+G/+A	eettcc
	4-9	+C/+T	−G (×7)/−8 (×3)	+G/+G	eettcc
	4-10	+G/+G	+T (×3)/−G (×7)	0 (×13)//+G (×2)//+A (×1)//+T (×2)	eettcx
T1	#6	+A (×2)//−5 (×1)//−41 (×2)//−52 + 9 (×3)//−24 (×2)	0 (×4)//+G (×5)//−G (×8)//−22 + 16 (×5)	0/0	extxCC
T2	6-1	−52 + 9/−52 + 9	−22 + 16(×6)/−3(×5)	0 (×7)//+T (×1)//+A (×1)	eettcx
	6-2	+T/+T	−G (×10)//+G (×5)//−26 (×4)	0 (×15)//+A (×2)//+T (×1)//−14 + 4 (×1)//−11 (×2)	eetxcx or eettcx
	6-3	−52 + 9/−52 + 9	+T (×5)//+G (×3)//−G (×2)//−22 + 16 (×3)	+A (×5)/+A (×5)	eetxcc or eettcc
	6-4	−52 + 9/−52 + 9	−22 + 16/−22 + 16	+G/+A	eettcc
	6-5	−52 + 9/−52 + 9	−22 + 16 (×5)/+G (×3)	+A/+T	eettcc
	6-6	−41 (×5)//−24 (×3)//−30 + 17 (×2)//+A (×2)	−G (×8)//+G (×2)//−22 + 16 (×1)	+A (×1)/+T (×8)	extxcc or eettcc
	6-7	−4 (×16)/+G (×4)	−G/−G	−G (×9)/+C (×2)	eettcc
	6-8	+C (×10)/−49 (×10)	−G/−G	+T/+T	eettcc
	6-9	−52 + 9/−52 + 9	+A (×5)//−G (×2)//−22 + 16 (×1)	+A/+T	eetxcc or eettcc
	6-10	+A/+T	−G/−G	+T/+T	eettcc

‘+’ indicates insertion, ‘–’ indicates deletion, ‘0’ indicates no mutation (wild-type allele). The number following ‘+’ or ‘–’ indicates the number of bases inserted or deleted; if the number is 1, it is replaced with a specific base. Mutations were detected by direct sequencing of PCR products or sequencing of cloned PCR products. Two types of mutations from direct sequencing of PCR products were obtained based on double-peaks on a chromatograph. When mutations were detected by sequencing of cloned PCR products, the number of the same type of mutation is indicated in parentheses. Two alleles (in WT, homozygous or biallelic mutants, or heterozygous mutants) are separated by ‘/’, whereas more than two alleles (in mosaic plants, underlined) are separated by ‘//’ between two alleles. For genotypes, E/T/C corresponds to the wild-type *ETC2*/*TRY*/*CPC* gene, e/t/c corresponds to *etc2*/*try*/*cpc* mutant gene, x corresponds to multiple alleles, resulting in mosaic plants

### Functional comparisons of 12 combinations of eight promoters and two terminators

**Fig. 3:**
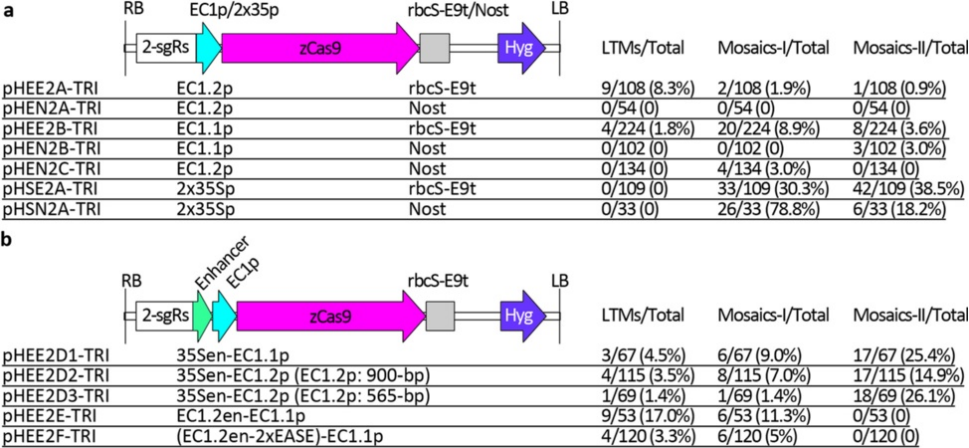
Structural and functional comparisons of twelve combinations of eight promoters and two terminators. **a** Seven combinations of *EC1.1*, *EC1.2*, or 2x35S promoters and *rbcS E9* terminator (rbcS-E9t) or *nos* terminator (Nost). The pHEN2A-TRI and pHEN2C-TRI constructs have the same combination but different vector backbones: pGreen for the former and pCambia for the latter. The data for pHSN2A-TRI come from the publication and p2gR-TRI-A is renamed pHSN2A-TRI in this paper [26]. **b** Five combinations of five fusion promoters and the *rbcS E9* terminator. Physical maps of the T-DNAs of seven (a) or five (b) CRISPR/Cas9 binary vectors are indicated. For each binary vector, the vector name, the promoter, the terminator, and the mutation frequencies of T1 transgenic plants are indicated at the same row under the maps. See Fig. 1 for RB/LB, *zCas9*, 2-sgRs, and Hyg. EC1p, EC1.1p or EC1.2p; 35Sen, CaMV 35S enhancer; EC1.2en, enhancer from EC1.2 promoter; LTM, likely triple mutant; Total, total number of T1 plants; Mosaics-I, type I mosaic plants with strong phenotypes indistinguishable from the double mutants; Mosaics-II, type II mosaic plants with the phenotypes appearing only in some parts of the whole plants. The ratios of T1 plants with the mutations (LTMs, Mosaics-I, or Mosaics-II) to total number of T1 plants are indicated

In an attempt to improve the efficiency of generating T1 homozygous mutants, we first tested another egg-cell specific promoter, using the promoter from *EC1.1*, and then we tested *EC1.2* or *EC1.1* promoters fused with enhancers (Fig. 3). Similar to our tests of the *EC1.2* promoter, we also tested two combinations of the *EC1.1* promoter with the *rbcS E9* terminator (pHEE2B-TRI) or *nos* terminator (pHEN2B-TRI) to further examine the effects of terminators on mutation efficiencies (Fig. 3a). We obtained 32 plants with observable mutations out of 224 T1 EC1.1p:zCas9-rbcS_E9t transgenic lines (Additional file 4: Figure S7). However, most plants with observable mutations seemed to be likely double mutants or mosaics, and only four plants seemed to be likely triple mutants (Additional file 4: Figure S7), suggesting that the *EC1.1* promoter is less egg cell-specific than the *EC1.2* promoter. The existence of a high ratio of mosaics means that the likely triple mutants (1.8 %) from EC1.1p:zCas9-rbcS_E9t transgenic lines are more likely to be phenotypically severe mosaics. We obtained only three mosaic plant out of 102 T1 EC1.1p:zCas9-Nost transgenic plants (Fig. 3a), demonstrating for the third time that the *rbcS E9* terminator performed much better than the *nos* terminator. To exclude the possibility that the pGreen backbone of pHEN2A-TRI and pHEN2B-TRI was the reason for the low mutation efficiencies, we constructed pHEN2C-TRI by replacing the *rbcS E9* terminator of pCambia1300-derived pHEE2A-TRI with the *nos* terminator (Fig. 3a). We obtained only four likely double mutants out of 134 T1 EC1.2p:zCas9-Nost (pCambia) lines, demonstrating for the fourth time that the *rbcS E9* terminator performed much better than the *nos* terminator, and the effects of the terminators were independent of the backbones of the binary vectors.

In our previous work, we demonstrated that constitutive overexpression of *zCas9* driven by the double 35S promoter in T1 2x35Sp:zCas9-Nost transgenic lines (using construct p2gR-TRI-A, renamed pHSN2A-TRI in this paper) efficiently produced mutations for *TRY*, *CPC*, and *ETC2*, but all the mutants were mosaics [26]. Since EC1p/rbcS-E9t combinations (pHEE2A-TRI and pHEE2B-TRI) performed much better than EC1p/Nost combinations (pHEN2A-TRI, pHEN2B-TRI, and pHEN2C-TRI), we reasoned that the 2x35Sp/rbcS-E9t combination (pHSE2A-TRI) would perform much better than the 2x35Sp/Nost combination (pHSN2A-TRI). We constructed pHSE2A-TRI (Fig. 3a), and obtained 109 T1 2x35Sp:zCas9-rbcS_E9t transgenic lines. None of the T1 lines are likely triple mutants (Fig. 3a), demonstrating again that almost all mutants produced from the T1 2x35S:Cas9 transgenic lines are mosaics. The ratios of mutants from T1 2x35Sp:zCas9-rbcS_E9t lines with strong (30.3 %) or observable (68.8 %) phenotypes to total number of T1 lines are much lower than those from T1 2x35Sp:zCas9-Nost lines (78.8 % and 97.0 %, respectively). These results demonstrated that 2x35Sp/rbcS-E9t combination did not perform much better than 2x35Sp/Nost combination, suggesting that in vegetative cells, the *nos* terminator seemed to work better than the *rbcS-E9* terminator. Considering statistical errors (for example, due to insufficient sample population for 2x35Sp:zCas9-Nost transgenic lines), another possibility is that *zCas9* mRNA stability is not as important for strong constitutive promoters as it is for egg cell-specific promoters.

To determine whether the 35S enhancer could increase the expression driven by the egg cell-specific promoters, we constructed three fusion promoters by fusing the 35S enhancer with the egg cell-specific promoters and then generated transgenic lines for the analysis of these fusion promoters’ activities (Fig. 3b). The ratio (26/67, 38.8 %) of 35Sen-EC1.1p:zCas9 plants with observable mutations to the total number of T1 transgenic lines was much higher than that (32/224, 14.3 %) of EC1.1p:zCas9-rbcS_E9t plants with observable mutations (Fig. 3b). In comparison with the ratio for T1 EC1.2p:zCas9-rbcS_E9t transgenic lines, the ratios of plants with observable mutations to total numbers of T1 35Sen-EC1.2p(900-bp):zCas9 or 35Sen-EC1.2p(565-bp):zCas9 transgenic lines greatly increased – 11.1 % (12/108), 25.2 % (29/115), and 29.0 % (20/69) for the three transgenic lines, respectively – whereas the ratios of likely triple mutants decreased (8.3 %, 3.5 %, and 1.4 % for the three transgenic lines, respectively) (Fig. 3b). These results demonstrated that the CaMV 35S enhancer increased the expression of *EC1.1* or *EC1.2* promoters but not in an egg cell-specific way. Thus, the CaMV 35S enhancer is not suitable for improving the EPC CRISPR/Cas9 system, which is consistent with our notion that the CaMV 35S promoter has weak activity in egg cells and one-cell stage embryos.

To determine whether the enhancer from the *EC1.2* promoter or EASE [53] could improve the performance of the EPC CRISPR/Cas9 system, we constructed another two fusion promoters by fusing the enhancer from the *EC1.2* promoter (EC1.2en), or EC1.2en plus double EASE enhancers (EC1.2en-2xEASE), with the *EC1.1* promoter (Fig. 3b). The ratio (17.0 %) of EC1.2en-EC1.1p:zCas9-rbcS_E9t plant-derived likely triple mutants to total number of T1 transgenic lines greatly increased (Fig. 3b, Additional file 4: Figure S8) in comparison with those for the EC1.2p/EC1.1p:zCas9-rbcS_E9t plant-derived mutants (8.3 % and 1.8 %, respectively, Fig. 3a). The ratio (28.3 %) of EC1.2en-EC1.1p:zCas9-rbcS_E9t plants with observable mutations to total number of T1 transgenic lines also greatly increased (Fig. 3b, Additional file 4: Figure S8) in comparison with those for the EC1.2p/EC1.1p:zCas9-rbcS_E9t plants with observable mutations (11.1 % and 14.3 %, respectively, Fig. 3a). These results demonstrated that the EC1.2en-EC1.1p fusion promoter performed much better than the single *EC1.2* or *EC1.1* promoters, and the enhancer from the *EC1.2* promoter significantly improved egg cell-specificity and expression strength of *EC1.1* promoter. Unexpectedly, when we added double EASE enhancers into the EC1.2en-EC1.1p fusion promoter, the resultant fusion promoter caused lower mutation efficiency: only 8.3 % (10/120) EC1.2en-2xEASE-EC1.1p:zCas9-rbcS_E9t plants harbor the observable mutations (Fig. 3b, Additional file 4: Figure S9). These results suggested that *EC1* and EASE have different mechanisms for egg cell-specific expression, and the two mechanisms seem to be antagonistic.

## Discussion

For most plants, including crops, genetic transformation is usually performed with embryogenic callus cells induced during tissue culture. Recent studies have demonstrated that embryogenic cell-derived transgenic lines expressing the CRISPR/Cas9 system could be homozygous mutant for edited target genes in the first generation [26–28, 33]. For *Arabidopsis*, the only plant species currently compatible with efficient, *in planta* transformation, most modifications detected in first-generation CRISPR/Cas9 transgenic lines were only somatic mutations [20, 25, 26, 29, 30, 34, 35]. Therefore it was proposed that screening of heritable mutations should be performed in the T2 or subsequent generations [35]. However, we reasoned that the excess of mosaic plants and lack of homozygous mutants was probably due to the low expression level of *Cas9* driven by constitutive promoters (usually the 35S promoter) in egg cells and one-cell stage embryos. Due to the stability of Cas9 mRNA and Cas9 protein, CRISPR/Cas9 specifically expressed in egg cells should function in one-cell embryos, allowing the creation of homozygous or bi-allelic T1 mutants. Consistent with this notion, in the current study, we used an egg cell-specific promoter to express *Cas9* and succeeded in creating T1 homozygous (or bi-allelic) mutants harboring two or three target genes that were modified simultaneously. More importantly, since approximately 8.3 % and 13.1 % of T1 plants are non-mosaic triple and double mutants for the representative three and two target genes, respectively, we demonstrated that mutation efficiencies of the system would be sufficient for producing customized *Arabidopsis* mutants. Although some mosaics still formed in T1 and T2 plants due to *Cas9* mRNA and/or protein stability (Table 3, Additional file 2: Tables S1 and S2), these mosaics were derived from early embryos (probably two- or four-celled embryos) and were thus stable in terms of the types and patterns of mutations (Additional file 2: Table S2). Therefore, some mosaic plants harboring more than two mutant alleles but lacking wild-type alleles are equivalent to homozygous (or bi-allelic) mutants in phenotypes or gene-specific traits. By contrast, the mutation types and patterns of the mosaic plants resulting from 35Sp:Cas9 transformation were usually unstable and variable throughout development and in different tissues or organs. So, even if some mosaics would be unavoidable to be produced from EC1.2p:Cas9, these mosaics could produce much higher ratios of homozygous (or bi-allelic) mutant progeny harboring much more highly predictable mutation types than mosaics from 35S:Cas9 could do. Therefore, the mosaics, if unavoidable, from EC1.2p:Cas9 or EC1.2en-EC1.1p:Cas9 are more useful for screening for homozygous mutant progeny, especially non-transgenic homozygous mutants in T2 generation, than those from 35S:Cas9 are (Additional file 3: Figure S4).

Ma *et al.* [54] recently reported that using 2x35Sp:Cas9p they obtained about 8.5 % non-mosaic T1 mutations (10 out of 118 sequenced target sites). Since these sequenced target sites involved six target sites in four genes (three targets in the same gene and three targets in three other genes) of about 100 T1 lines, this means that they obtained approximately 9.0 % (9/100) non-mosaic T1 mutants. They obtained one T1 mutant, out of 14 T1 lines, with non-mosaic, simultaneous mutations of two target sites of the same gene. However, they failed to obtain the mutants (0/14) with simultaneous mutations of three target sites. These results suggest that 2x35Sp:Cas9 transgenic lines have much lower mutation efficiencies for the generation of T1 homozygous or biallelic mutants for multiple target genes than EC1.2p:Cas9 transgenic lines, which is consistent with our results.

In this study, to evaluate mutation efficiencies and determine the size of the T1 population needed for screening for homozygous mutants, we used screenable phenotypes for the identification of the triple or double mutants. In practical applications, the genes-of-interest might have no convenient, visible phenotype. One potential, low-cost strategy for identifying mutants is to select, as far as possible, targets with cleavage sites located within restriction enzyme sites. In this case, users can conduct primary screening by restriction enzyme digestion analysis (Additional file 3: Figure S5), as the occurrence of a mutation should disrupt the restriction enzyme site [26]. Alternatively, users could conduct primary screening by T7E1 or Surveyor assay [8, 22]. Then, users can perform sequencing analysis, based on three strategies, of primarily screened lines that likely are bi-allelic mutants (Additional file 3: Figure S5). One strategy is direct sequencing purified PCR fragments spanning target sites using primers within PCR fragments. For homozygous (or bi-allelic) mutations with a one base pair insertion or deletion, this strategy would work well. Sequencing of individual clones of PCR fragments and deep sequencing of PCR fragments can also be used to identify non-mosaic, mutant plants. However, the high frequencies of mutations induced by this method will also allow users to identify non-mosaic mutants by using the simplest and most effective (but also expensive) method, deep sequencing of PCR fragments spanning target sites from 25–50 T1 transgenic lines.

Some targets may have much lower mutation efficiencies than others, so we suggest selecting three sets of targets, with two sets as backups to avoid being delayed by possibly recalcitrant targets (Additional file 3: Figure S5). Construction of a binary vector harboring two sgRNAs is very simple and only a single additional PCR fragment is required for the cloning system [26]. Therefore, even for targeted mutation of a single gene, we suggest always constructing a binary vector that harbors two sgRNAs targeting two sites in the same gene. Thus, with two backup vectors, this method provides sextuple assurance of getting targeted mutations of a single gene.

A critical finding in this study is that one of the key factors determining our success with the EPC CRISPR/Cas9 system was the presence of a suitable terminator. T1 and T2 plants with observable mutations (including likely triple/double mutants and mosaics) were infrequent when these plants were derived from EC1.2p:zCas9-Nost transgenic lines, which used the *nos* terminator. By contrast, an average of 24.8 % T2 plants derived from EC1.2p:zCas9-rbcS_E9t, which used the *rbcS-E9* terminator, were likely triple mutants (Table 2). Indeed, while the use of the same promoter with different terminators has been shown to sometimes result in significantly different levels of protein accumulation [51], the large difference in mutation efficiencies observed between the two terminators in the current study was unexpected. Comparison of mutation frequencies of additional constructs (pHEE2B-TRI and pHEN2B-TRI, or pHEE2A-TRI and pHEN2C-TRI) provided additional proof that the *rbcS E9* terminator performed much better than the *nos* terminator (Fig. 3). These results suggest that the presence of the proper terminator is a key factor in stabilizing *Cas9* mRNA in egg cells, and different terminators have significantly different effects on *Cas9* mRNA stability.

CRISPR/Cas9-based multiplex genome editing requires multiple sgRNAs and maintaining appropriate concentrations of each variant of sgRNA-Cas9 complex in a cell for target search according to the target recognition mechanism [55]. Since methods have been developed to assemble multiple sgRNAs [26, 37, 54, 56, 57], highly efficient expression of multiple sgRNAs in a cell has not been a problem. However, co-existence of multiple sgRNAs in a cell would dilute the concentration of each variant of the sgRNA-Cas9 complex harboring a specific target sequence. Thus, although the total concentration of all sgRNA-Cas9 complexes may remain stable, the functional concentration of each variant of the sgRNA-Cas9 complex would decrease in inverse proportion to the numbers of sgRNA variants or target sites. Thus, it is important to express Cas9 more efficiently to increase the concentration of each variant of the sgRNA-Cas9 complex and thus enhance the efficiency of multiplex genome editing. Consistent with this notion, our results demonstrated that mutation efficiencies for multiple targets could be greatly increased by using not only appropriate promoters to drive the expression of *Cas9* but also appropriate terminators to stabilize *Cas9* mRNA. These observations should facilitate development or improvement of genome editing methods for the generation of non-mosaic mutants for multiple target genes in other organisms, especially through specific expression of CRISPR/Cas9 in germline cells, gametes, or one-cell stage embryos [58]. Although currently *Arabidopsis* is the only species amenable to *in planta* transformation method with high efficiency, along with the development of *in planta* transformation for other plants, it is possible that the egg cell-specific promoter-controlled CRISPR/Cas9 system or similar strategies will be very useful for more plant species.

Localized egg-cell expression of a ZFN was previously employed for targeted editing of the *Arabidopsis* genome [59]. In this case, the EASE:QQR-ZFN expression cassette was used; EASE is an enhancer sequence that specifically regulates gene expression in the egg apparatus of *Arabidopsis*, and QQR-ZFN is a well-characterized ZFN that functions *in planta* [53, 59]. However, the reported mutation frequencies were not high enough for practical application. No mutations were detected in T1 EASE:QQR-ZFN plants, and the mutation frequency of T2 plants was only 0.078 % (7/9000) in a GUS staining assay and only 0.27 % (1/366) in a PCR-based assay. Even if the actual mutation rate were underestimated, the mutation frequency of T2 plants was not much higher than 0.5 % for the single target gene. By contrast, in our EC1.2p:Cas9-based system, approximately 8.3 % of the T1 plants and an average of 24.8 % of the T2 plants were likely triple mutants (Table 3). These results might reflect the differences between CRISPR/Cas9 and ZFN, between EC1.2p and EASE, and/or between the terminators used in the two cassettes.

Although the mutation efficiencies that can be obtained using the *EC1.2* promoter-controlled CRISPR/Cas9 system are high enough to allow researchers to customize their own *Arabidopsis* mutants, higher target gene editing efficiencies can be anticipated. As an example, the fusion promoter from two egg cell-specific genes *EC1.2* and *EC1.1* resulted in much higher efficiency of mutation compared with the single promoters. By fusing more enhancers from the *EC1* genes (including *EC1.1*–*EC1.5*) to the *EC1.1* or *EC1.2* promoter [50], stronger and more specific expression of *Cas9* in egg cells and one-cell stage embryos could be anticipated. In addition, by using more effective terminators than *rbcS E9* terminator, the EPC CRISPR/Cas9 system could be further improved. The optimized combinations of fusion promoters and terminators will greatly enhance the mutation efficiencies. It has been shown that, after transfection by *Agrobacterium*, the ratio (7 %) of ovules demonstrating transient expression to total number of ovules examined was much higher than ratio (0.44 %) of ovules developing into stable transgenic seeds to total number of ovules/seeds examined [59]. Thus, this enhanced system would allow us to create non-transgenic, gain-of-function T1 mutants via homologous recombination mediated by the EPC CRISPR/Cas9 system, which is currently a formidable challenge [35]. Finally, with the development of high-throughput sequencing, deep sequencing of large batches of PCR products will become affordable and the time required to identify targeted gene modifications will be further shortened.

## Conclusions

Probing gene function and examining gene interactions requires the generation of single, double, and multiple mutants in different combinations. However, in plants, generation of these mutants requires screening of banks of existing mutants, followed by laborious and time-consuming crossing and screening for multiple mutants. New genome-editing methods, such as the CRISPR/Cas9 system, can be used to generate targeted gene modifications in *Arabidopsis*; however, almost all first-generation CRISPR/Cas9 transgenic *Arabidopsis* plants have been mosaic for the targeted genes. This study demonstrates that specifically expressing *Cas9* in egg cells and one-cell stage embryos enables the creation of homozygous or biallelic T1 mutants for multiple target genes with high efficiency: 2 of 24 (8.3 %) of the T1 plants were homozygous or biallelic *cpc try etc2* triple mutants. Moreover, 12 of the 24 T1 plants gave rise to homozygous triple mutants in the T2 generation. The segregation ratio of likely triple mutants to total T2 plants was over 20 % for all 12 T1 lines and averaged 24.8 % for all 24 T1 lines. We also generated *chli1 chli2* homozygous or biallelic double mutants with a ratio of 13.1 % (13/99) in T1 generation. Comparisons of 12 combinations of eight promoters and two terminators found that the efficiency of the egg cell-specific promoter-controlled (EPC) CRISPR/Cas9 system depended on the presence of a suitable terminator, and the fusion promoter from two egg cell-specific genes *EC1.2* and *EC1.1* resulted in much higher efficiency of mutation in the T1 generation compared with the single promoters. This system provides a rapid, cost-effective way to create new mutant populations and multi-gene mutants in *Arabidopsis*. This study also presented a route to optimize the EPC CRISPR/Cas9 system.

## Methods

### Vector construction

Detailed descriptions of the vector construction are provided in Additional file 5: Methods S1. All primers used in this study are listed in Additional file 2: Table S3. The vector sequences are provided in Additional file 6.

### Generation of transgenic *Arabidopsis* plants and analysis of mutations

We transformed the pHEE2A/B/D1/D2/D3/E/F-TRI, pHEN2C-TRI, pHSE2A-TRI, and pHEE2A-CHLI constructs into *Agrobacterium* strain GV3101, and transformed pHEN2A/B-TRI into GV3101/pSoup [26]. We transformed *Arabidopsis* Col-0 wild-type plants via the floral dip method [45]. We screened the collected seeds from the T0 plants on MS plates containing 25 mg/L hygromycin, and transplanted the resistant seedlings (T1) to soil. We extracted genomic DNA from T1 transgenic plants grown in soil. To analyze mutations of *TRY*, *CPC*, and *ETC2*, we amplified fragments surrounding the target sites of *TRY*, *CPC*, or *ETC2* by PCR using gene-specific primers TRY-IDF0/R0, CPC-IDF0/R0, or ETC2-IDF0/R0, respectively [26]. We submitted purified PCR products for direct sequencing with primers TRY/CPC/ETC2-seqF [26] located within the PCR fragments. To analyze possible mutations of potential off-target sites of *TRY*, *CPC*, and *AT5G50230* of the sgRNA targeting *ETC2*, we amplified fragments surrounding the off-target sites by PCR using gene-specific primers TRY-off-IDF/R, CPC-off-IDF2/R, or 5G50230-off-IDF/R, respectively. We submitted purified PCR products for direct sequencing (as opposed to sequencing of individual clones of PCR products) with primers TRY/CPC/5G50230-off-seqF located within the PCR fragments. To analyze mutations of *CHLI1* and *CHLI2*, we amplified fragments surrounding the target sites of *CHLI1* or *CHLI2* by PCR using gene-specific primers CHLI1-IDF/R or CHLI2-IDF/R, respectively. We submitted purified PCR products for direct sequencing with primers CHLI1/2-seqF located within the PCR fragments. We then cloned poorly sequenced PCR products, and submitted individual positive clones for sequencing using the T7 primer. To screen the segregated non-transgenic T2 plants, we first screened nine primer combinations, with three forward primers including zCas9-IDF3-2/-IDF5/-IDF6 (located at *zCas9*) and three reverse primers including rbcS_E9t-IDR/-IDR2 (located at *rbcS-E9* terminator) and lacp-IDF (located at the *lac* promoter of the vector backbone), for more specific primers (Additional file 2: Table S3). We obtained three more specific primer pairs, including zCas9-IDF3-2/rbcS_E9t-IDR2, zCas9-IDF5/lacp-IDF, and zCas9-IDF6/lacp-IDF, with wild-type genomic DNA serving as a negative control and genomic DNA from T1 transgenic plants serving as a positive control (Additional file 2: Table S3). We then performed counterselection PCR with the three primer pairs for screening of non-transgenic T2 plants.

## Additional files

Additional file 1: Figure S1.
*Arabidopsis* T1 likely triple mutants obtained from the second round of transformation. **Figure S2.** *Arabidopsis* T1 likely triple mutants obtained from the third round of transformation. **Figure S3.** *Arabidopsis* T1 homozygous double mutants obtained via EPC CRISPR/Cas9. Additional file 2: Table S1. Mutation analysis of T1 albino mutants. **Table S2.** Supposed Cas9 protein dynamics during early embryo development. **Table S3.** Primers used in this study. Additional file 3: Figure S4. Flow chart for the creation of *Arabidopsis* T1 homozygous mutants via EPC CRISPR/Cas9. **Figure S5.** Strategy for screening for T1 bi-allelic mutants with no observable phenotypes. **Figure S6.** The triple mutant can be differentiated from the double mutant. Additional file 4: Figure S7. Thirty-two out of 224 T1 pHEE2B-TRI transgenic plants harbor observable mutations. **Figure S8.** Fifteen out of 53 T1 pHEE2E-TRI transgenic plants harbor observable mutations. **Figure S9.** Ten out of 120 T1 pHEE2F-TRI transgenic plants harbor observable mutations. Additional file 5:
**Methods S1. Vector construction.**
Additional file 6:
**Vector maps and sequences.**

## References

[1] McCourt P, Benning C (2010). Arabidopsis: a rich harvest 10 years after completion of the genome sequence. Plant J.

[2] O’Malley RC, Ecker JR (2010). Linking genotype to phenotype using the Arabidopsis unimutant collection. Plant J.

[3] Belhaj K, Chaparro-Garcia A, Kamoun S, Nekrasov V (2013). Plant genome editing made easy: targeted mutagenesis in model and crop plants using the CRISPR/Cas system. Plant Methods.

[4] Pennisi E (2013). The CRISPR craze. Science.

[5] Voytas DF (2013). Plant genome engineering with sequence-specific nucleases. Annu Rev Plant Biol.

[6] Segal DJ (2013). Bacteria herald a new era of gene editing. Elife.

[7] Hsu PD, Lander ES, Zhang F (2014). Development and applications of CRISPR-Cas9 for genome engineering. Cell.

[8] Cong L, Ran FA, Cox D, Lin S, Barretto R, Habib N (2013). Multiplex genome engineering using CRISPR/Cas systems. Science.

[9] Jinek M, Chylinski K, Fonfara I, Hauer M, Doudna JA, Charpentier E (2012). A programmable dual-RNA-guided DNA endonuclease in adaptive bacterial immunity. Science.

[10] Mali P, Yang L, Esvelt KM, Aach J, Guell M, DiCarlo JE (2013). RNA-guided human genome engineering via Cas9. Science.

[11] Hwang WY, Fu Y, Reyon D, Maeder ML, Tsai SQ, Sander JD (2013). Efficient genome editing in zebrafish using a CRISPR-Cas system. Nat Biotechnol.

[12] Jao LE, Wente SR, Chen W (2013). Efficient multiplex biallelic zebrafish genome editing using a CRISPR nuclease system. Proc Natl Acad Sci U S A.

[13] Li D, Qiu Z, Shao Y, Chen Y, Guan Y, Liu M (2013). Heritable gene targeting in the mouse and rat using a CRISPR-Cas system. Nat Biotechnol.

[14] Li W, Teng F, Li T, Zhou Q (2013). Simultaneous generation and germline transmission of multiple gene mutations in rat using CRISPR-Cas systems. Nat Biotechnol.

[15] Wang H, Yang H, Shivalila CS, Dawlaty MM, Cheng AW, Zhang F (2013). One-step generation of mice carrying mutations in multiple genes by CRISPR/Cas-mediated genome engineering. Cell.

[16] Niu Y, Shen B, Cui Y, Chen Y, Wang J, Wang L (2014). Generation of gene-modified cynomolgus monkey via Cas9/RNA-mediated gene targeting in one-cell embryos. Cell.

[17] Li JF, Norville JE, Aach J, McCormack M, Zhang D, Bush J (2013). Multiplex and homologous recombination-mediated genome editing in Arabidopsis and Nicotiana benthamiana using guide RNA and Cas9. Nat Biotechnol.

[18] Nekrasov V, Staskawicz B, Weigel D, Jones JD, Kamoun S (2013). Targeted mutagenesis in the model plant Nicotiana benthamiana using Cas9 RNA-guided endonuclease. Nat Biotechnol.

[19] Shan Q, Wang Y, Li J, Zhang Y, Chen K, Liang Z (2013). Targeted genome modification of crop plants using a CRISPR-Cas system. Nat Biotechnol.

[20] Feng Z, Zhang B, Ding W, Liu X, Yang DL, Wei P (2013). Efficient genome editing in plants using a CRISPR/Cas system. Cell Res.

[21] Jiang W, Zhou H, Bi H, Fromm M, Yang B, Weeks DP (2013). Demonstration of CRISPR/Cas9/sgRNA-mediated targeted gene modification in Arabidopsis, tobacco, sorghum and rice. Nucleic Acids Res.

[22] Mao Y, Zhang H, Xu N, Zhang B, Gou F, Zhu JK (2013). Application of the CRISPR-Cas system for efficient genome engineering in plants. Mol Plant.

[23] Miao J, Guo D, Zhang J, Huang Q, Qin G, Zhang X (2013). Targeted mutagenesis in rice using CRISPR-Cas system. Cell Res.

[24] Xie K, Yang Y (2013). RNA-guided genome editing in plants using a CRISPR-Cas system. Mol Plant.

[25] Jiang W, Yang B, Weeks DP (2014). Efficient CRISPR/Cas9-mediated gene editing in Arabidopsis thaliana and inheritance of modified genes in the T2 and T3 generations. PLoS One.

[26] Xing HL, Dong L, Wang ZP, Zhang HY, Han CY, Liu B (2014). A CRISPR/Cas9 toolkit for multiplex genome editing in plants. BMC Plant Biol.

[27] Zhou H, Liu B, Weeks DP, Spalding MH, Yang B (2014). Large chromosomal deletions and heritable small genetic changes induced by CRISPR/Cas9 in rice. Nucleic Acids Res.

[28] Zhang H, Zhang J, Wei P, Zhang B, Gou F, Feng Z (2014). The CRISPR/Cas9 system produces specific and homozygous targeted gene editing in rice in one generation. Plant Biotechnol J.

[29] Fauser F, Schiml S, Puchta H (2014). Both CRISPR/Cas-based nucleases and nickases can be used efficiently for genome engineering in Arabidopsis thaliana. Plant J.

[30] Schiml S, Fauser F, Puchta H (2014). The CRISPR/Cas system can be used as nuclease for in planta gene targeting and as paired nickases for directed mutagenesis in Arabidopsis resulting in heritable progeny. Plant J.

[31] Gao J, Wang G, Ma S, Xie X, Wu X, Zhang X (2015). CRISPR/Cas9-mediated targeted mutagenesis in Nicotiana tabacum. Plant Mol Biol.

[32] Johnson RA, Gurevich V, Filler S, Samach A, Levy AA (2015). Comparative assessments of CRISPR-Cas nucleases’ cleavage efficiency in planta. Plant Mol Biol.

[33] Brooks C, Nekrasov V, Lippman ZB, Van Eck J (2014). Efficient gene editing in tomato in the first generation using the clustered regularly interspaced short palindromic repeats/CRISPR-associated9 system. Plant Physiol.

[34] Hyun Y, Kim J, Cho SW, Choi Y, Kim JS, Coupland G (2015). Site-directed mutagenesis in Arabidopsis thaliana using dividing tissue-targeted RGEN of the CRISPR/Cas system to generate heritable null alleles. Planta.

[35] Feng Z, Mao Y, Xu N, Zhang B, Wei P, Yang DL (2014). Multigeneration analysis reveals the inheritance, specificity, and patterns of CRISPR/Cas-induced gene modifications in Arabidopsis. Proc Natl Acad Sci U S A.

[36] Upadhyay SK, Kumar J, Alok A, Tuli R (2013). RNA-guided genome editing for target gene mutations in wheat. G3 (Bethesda).

[37] Gao Y, Zhao Y (2014). Self-processing of ribozyme-flanked RNAs into guide RNAs in vitro and in vivo for CRISPR-mediated genome editing. J Integr Plant Biol.

[38] Liang Z, Zhang K, Chen K, Gao C (2014). Targeted mutagenesis in Zea mays using TALENs and the CRISPR/Cas system. J Genet Genomics.

[39] Li JF, Zhang D, Sheen J (2014). Cas9-based genome editing in Arabidopsis and tobacco. Methods Enzymol.

[40] Wang Y, Cheng X, Shan Q, Zhang Y, Liu J, Gao C (2014). Simultaneous editing of three homoeoalleles in hexaploid bread wheat confers heritable resistance to powdery mildew. Nat Biotechnol.

[41] Shan Q, Wang Y, Li J, Gao C (2014). Genome editing in rice and wheat using the CRISPR/Cas system. Nat Protoc.

[42] Sugano SS, Shirakawa M, Takagi J, Matsuda Y, Shimada T, Hara-Nishimura I (2014). CRISPR/Cas9-mediated targeted mutagenesis in the liverwort Marchantia polymorpha L. Plant Cell Physiol.

[43] Jia H, Wang N (2014). Targeted genome editing of sweet orange using Cas9/sgRNA. PLoS One.

[44] Xu R, Li H, Qin R, Wang L, Li L, Wei P (2014). Gene targeting using the Agrobacterium tumefaciens-mediated CRISPR-Cas system in rice. Rice (N Y).

[45] Clough SJ, Bent AF (1998). Floral dip: a simplified method for Agrobacterium-mediated transformation of Arabidopsis thaliana. Plant J.

[46] Ye GN, Stone D, Pang SZ, Creely W, Gonzalez K, Hinchee M (1999). Arabidopsis ovule is the target for Agrobacterium in planta vacuum infiltration transformation. Plant J.

[47] Bechtold N, Jaudeau B, Jolivet S, Maba B, Vezon D, Voisin R (2000). The maternal chromosome set is the target of the T-DNA in the in planta transformation of Arabidopsis thaliana. Genetics.

[48] Desfeux C, Clough SJ, Bent AF (2000). Female reproductive tissues are the primary target of Agrobacterium-mediated transformation by the Arabidopsis floral-dip method. Plant Physiol.

[49] Steffen JG, Kang IH, Macfarlane J, Drews GN (2007). Identification of genes expressed in the Arabidopsis female gametophyte. Plant J.

[50] Sprunck S, Rademacher S, Vogler F, Gheyselinck J, Grossniklaus U, Dresselhaus T (2012). Egg cell-secreted EC1 triggers sperm cell activation during double fertilization. Science.

[51] Sarrion-Perdigones A, Vazquez-Vilar M, Palaci J, Castelijns B, Forment J, Ziarsolo P (2013). GoldenBraid 2.0: a comprehensive DNA assembly framework for plant synthetic biology. Plant Physiol.

[52] Bae S, Park J, Kim JS (2014). Cas-OFFinder: a fast and versatile algorithm that searches for potential off-target sites of Cas9 RNA-guided endonucleases. Bioinformatics.

[53] Yang W, Jefferson RA, Huttner E, Moore JM, Gagliano WB, Grossniklaus U (2005). An egg apparatus-specific enhancer of Arabidopsis, identified by enhancer detection. Plant Physiol.

[54] Ma X, Zhang Q, Zhu Q, Liu W, Chen Y, Qiu R et al. A Robust CRISPR/Cas9 system for convenient, high-efficiency multiplex genome editing in monocot and dicot plants. Mol Plant. 2015. 10.1016/j.molp.2015.04.007. 10.1016/j.molp.2015.04.007 25917172

[55] Sternberg SH, Redding S, Jinek M, Greene EC, Doudna JA (2014). DNA interrogation by the CRISPR RNA-guided endonuclease Cas9. Nature.

[56] Sakuma T, Nishikawa A, Kume S, Chayama K, Yamamoto T (2014). Multiplex genome engineering in human cells using all-in-one CRISPR/Cas9 vector system. Sci Rep.

[57] Xie K, Minkenberg B, Yang Y (2015). Boosting CRISPR/Cas9 multiplex editing capability with the endogenous tRNA-processing system. Proc Natl Acad Sci U S A.

[58] Ren X, Sun J, Housden BE, Hu Y, Roesel C, Lin S (2013). Optimized gene editing technology for Drosophila melanogaster using germ line-specific Cas9. Proc Natl Acad Sci U S A.

[59] Even-Faitelson L, Samach A, Melamed-Bessudo C, Avivi-Ragolsky N, Levy AA (2011). Localized egg-cell expression of effector proteins for targeted modification of the Arabidopsis genome. Plant J.

