# Supplementary material for: Egg cell-specific promoter-controlled CRISPR/Cas9 efficiently generates homozygous mutants for multiple target genes in Arabidopsis in a single generation
Source: Genome Biol. 2015 Jul 21;16(1):144. doi: 10.1186/s13059-015-0715-0 (PMC4507317; doi:10.1186/s13059-015-0715-0)
Supplement: Additional file 1: Figure S1. — Arabidopsis T1 likely triple mutants obtained from the second round of transformation. Figure S2. Arabidopsis T1 likely triple mutants obtained from the third round of transformation. Figure S3. Arabidopsis T1 homozygous double mutants obtained via EPC CRISPR/Cas9. [file 13059_2015_715_MOESM1_ESM.pdf]

Additional file 1: Figure S1

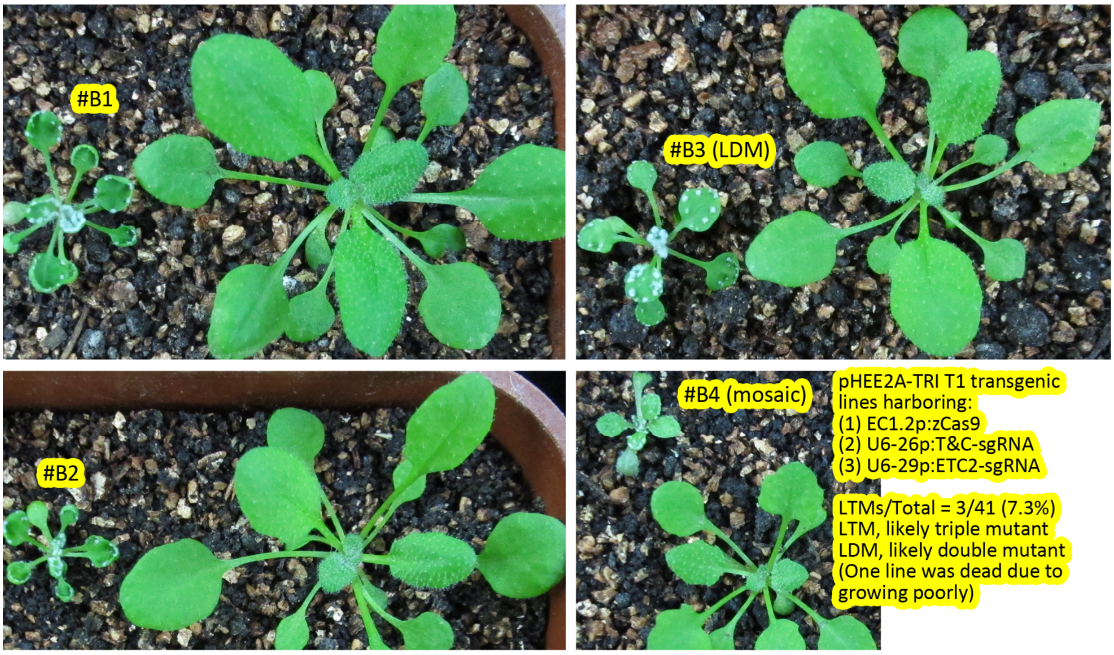

Figure S1. ***Arabidopsis*** **T1 likely triple mutants obtained from the second round of transformation.** Seeds from T0 plants were sown on MS medium containing 25 mg/L hygromycin, vernalized at 4°C for 3 days, and grown under long-day conditions (16 h light/8 h dark) at 22°C for 11 days. Hygromycin-resistant seedlings (T1) were transplanted to soil and allowed to grow for 26 days before photographing.

Additional file 2: Figure S2

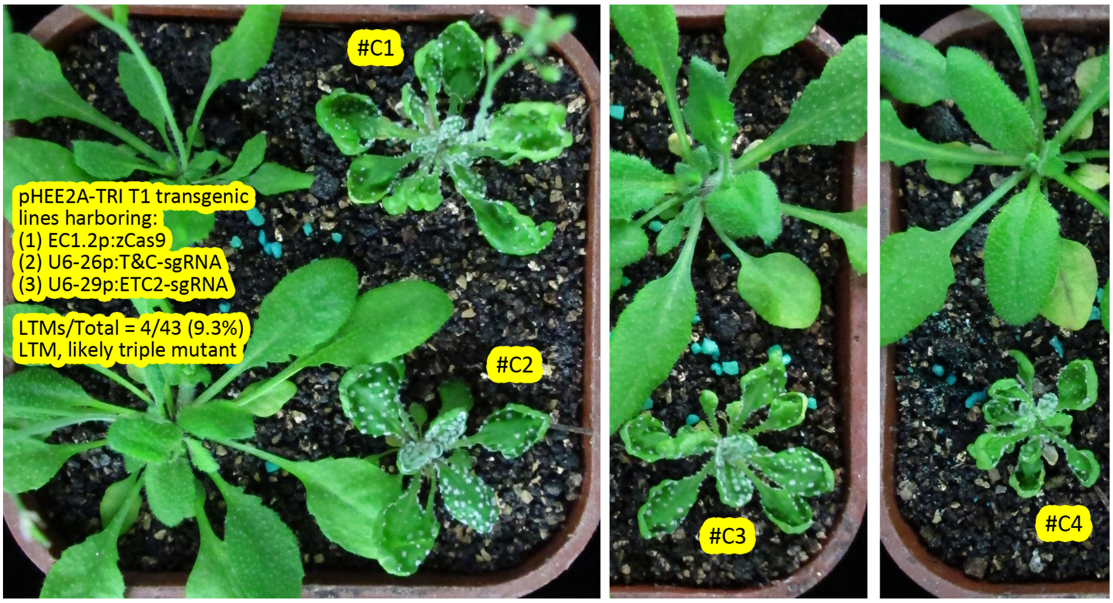

Figure S2. ***Arabidopsis*** **T1 likely triple mutants obtained from the third round of transformation.** Seeds from T0 plants were sown on MS medium containing 25 mg/L hygromycin, vernalized at 4°C for 3 days, and grown under long-day conditions (16 h light/8 h dark) at 22°C for 8 days. Hygromycin-resistant seedlings (T1) were transplanted to soil and allowed to grow for 32 days before photographing.

Additional file 2: Figure S3

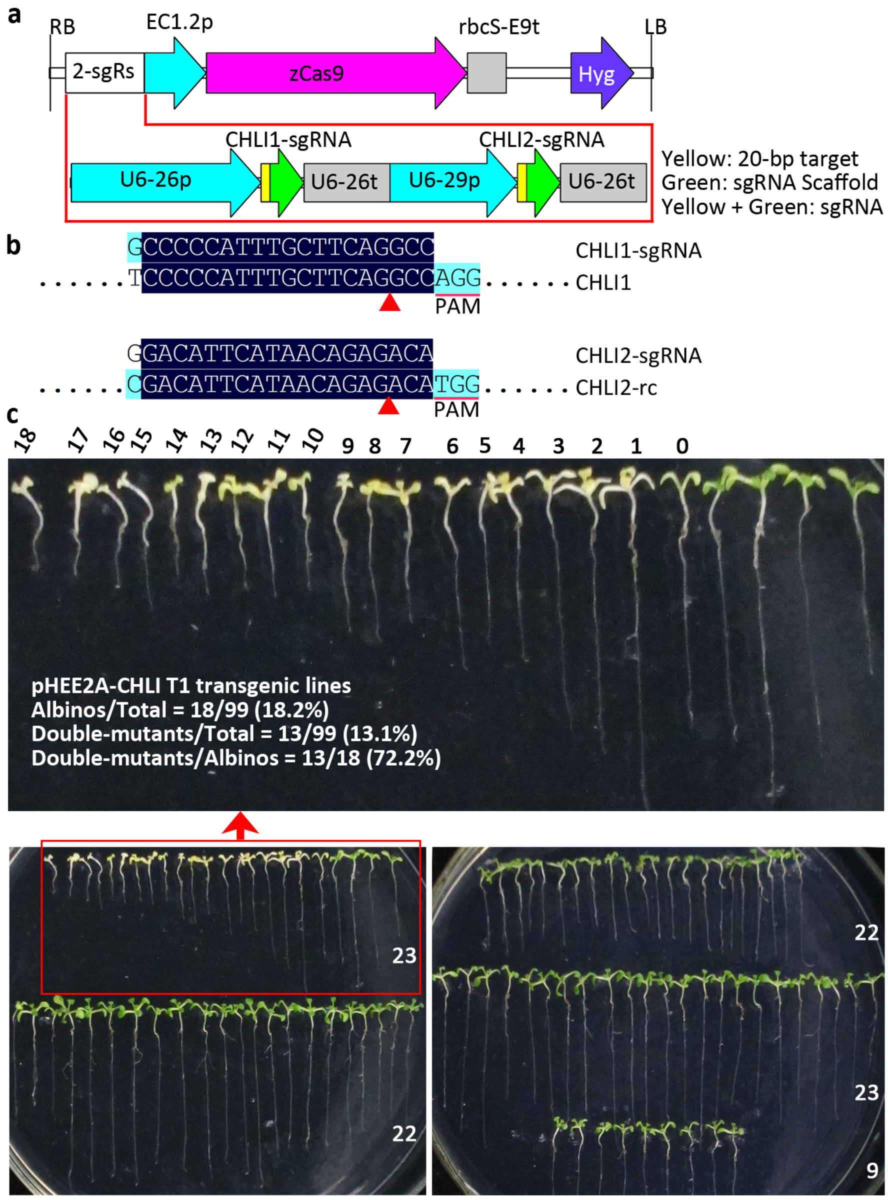

Figure S3. ***Arabidopsis*** **T1 homozygous double mutants obtained via EPC CRISPR/Cas9.** **(a)** Physical maps of the T-DNA of CRISPR/Cas9 binary vector carrying *Cas9* driven by the egg-cell specific *EC1.2* gene promoter and two sgRNA genes driven by Pol-III promoters U6-26p and U6-29p, respectively. See Figure 1 for RB, LB, EC1.2p, rbcS-E9t, 2-sgRs, zCas9, U6-26p, U6-29p, U6-26t, and *Hyg*. **(b)** The alignment of the sgRNA with its target genes and potential off-targets. Only aligned regions of interest are displayed. -rc, reverse complement. **(c)** Phenotypes of all transgenic seedlings from a single screening. Seeds from T0 plants were sown on MS medium containing 25 mg/L hygromycin, vernalized at 4°C for 3 days, and grown under long-day conditions (16 h light/8 h dark) at 22°C for 7 days. Hygromycin-resistant seedlings (T1) were transferred to two fresh MS plates and allowed to recover for 3 days before photographing. The number of seedlings in each row of a plate is indicated. Photographs of albino seedlings are enlarged and numbered.
