# Supplementary material for: Egg cell-specific promoter-controlled CRISPR/Cas9 efficiently generates homozygous mutants for multiple target genes in Arabidopsis in a single generation
Source: Genome Biol. 2015 Jul 21;16(1):144. doi: 10.1186/s13059-015-0715-0 (PMC4507317; doi:10.1186/s13059-015-0715-0)
Supplement: Additional file 2: Table S1. — Mutation analysis of T1 albino mutants. Table S2. Supposed Cas9 protein dynamics during early embryo development. Table S3. Primers used in this study. [file 13059_2015_715_MOESM2_ESM.pdf]

Additional file 2: Table S1

Table S1. Mutation analysis of T1 albino mutants

Line	*CHLI1* mutation	*CHLI2* mutation	Genotype
**0**	+G/+G	0/0	aaBB
**1**	+G/+T	+A/+C	aabb
**2**	+T (×2)/−7+5 (×4)	+C/+C	aabb
**3**	+T/+T	−13/−13	aabb
**4**	+A/+G	−T (×3)/−15 (×3)	aabb
**5**	n.a.	n.a.	n.a.
**6**	−8/−8	+C/+C	aabb
**7**	0 (x7)//-G (x9)//+A (x2) //-5 (x1)//-5 (x1)//-58 (x1)	−2 (×1)/+C (×5)	axbb
**8**	+C/+G	−C (×5)/+C (×1)	aabb
**9**	−G/−G	+C (×5)/−19+4 (×3)	aabb
**10**	+A/+G	−2 (×1)//−13 (×3)//+8 (×3)	aabx
**11**	−23 (×3)/+G (×4)	0 (×2)//−7 (×1)//−6+21 (×3)	aabx
**12**	+G/+G	0 (×12)/+A (×14)	aaBb or aabx
**13**	+G (×3)/+2 (×5)	−2 (×5)/−16+T (×1)	aabb
**14**	−22 (×5)/+G (×1)	0 (×1)//−T (×1)//−2 (×1)//+2 (×1)//−19+4 (×3)	aabx
**15**	n.a.	n.a.	n.a.
**16**	n.a.	n.a.	n.a.
**17**	−7 (×1)/+A (×5)	−15 (×4)/−51 (×2)	aabb
**18**	+G (×3)/−7 (×3)	+A/+A	aabb

"+" indicates insertion, "–" indicates deletion, "0" indicates no mutation (wild-type allele). The number following "+" or "–" indicates the number of bases inserted or deleted; if the number is 1, it is replaced with a specific base. Mutations were detected by direct sequencing of PCR products or sequencing of cloned PCR products. Two types of mutations from direct sequencing of PCR products were obtained based on double-peaks on a chromatograph. When mutations were detected by sequencing of cloned PCR products, the number of the same type of mutation is indicated in parentheses. Two alleles (in WT, homozygous or biallelic mutants, or heterozygous mutants) are separated by "/", whereas more than two alleles (in mosaic plants, underlined) are separated by "//" between two alleles. For genotypes, A/B corresponds to the wild-type *CHLI1*/*CHLI2* gene, a/b corresponds to *chli1*/*chli2* mutant gene, and x corresponds to multiple alleles resulting in a mosaic. n.a., not available.

Additional file 1: Table S2

Table S2. Supposed Cas9 protein dynamics during early embryo development

mRNA or protein content (molecules/cell)	EC	zygote	Embryo proper			
			1 cell	2 cells	4 cells	8 cells
***Cas9* mRNA**	1	1	1/2	1/4	1/8	1/16
**Translated Cas9 protein**	1	1	1/2	1/4	1/8	1/16
**Residual Cas9 protein**	0	0	1/2	1/2	3/8	1/4
**Total Cas9 protein**	1	1	1	3/4	1/2	5/16

The Cas9 protein dynamic levels during early embryo development are based on the suppositions that *Cas9* mRNA and Cas9 protein were sufficiently stable and translated Cas9 protein content was in proportion to mRNA content. The zygote divides into two daughter cells: an upper terminal cell and a lower basal cell. The terminal cell gives rise to the embryo proper that will form most of the mature embryo. By contrast, the basal cell forms the suspensor, which senesces after the heart stage and is not a functional part of the embryo in the mature seed. Thus, there are three one-cell stages at which CRISPR/Cas9 system functions: egg cell (EC), zygote and one-cell stage embryo proper. One-cell stage embryo proper comes from the mitosis of the zygote, therefore the content of *Cas9* mRNA or residual Cas9 protein is supposed to be half that of zygote. The total Cas9 protein is the sum of the newly translated Cas9 protein from mRNA and residual Cas9 protein from mother cell. Please note: the assumptions, on which this table is based, represent simplified or idealized conditions, which helps to better understand the effects of Cas9 mRNA and/or protein stability on mutations.

Additional file 1: Table S3

Table S3. Primers used in this study

Primer name	Primer sequence (5′→3′)
U6-26-HiF2	ATTATTATCAAGCTTCGACTTGCCTTCCGCAC
U6-26-XNR	CATTATTCTAGATTATCCATGGTATTGGTTTATCTCATCG
EC1.2p-NcF	ATTATTACCATGGACTTTGATAAATGTTCCTCGCTGAC
EC1.2p-NhR	ATTATTGCTAGCTTATTCTTTCTTTTTGGGGTT
EC1.1p-NcF	AATATTCCATGGAACGCCTATCATGAATTAGC
EC1.1p-XbR	AATATTATTCTAGATTTCTCAACAGATTGATAAGGTCG
EC1.1p-NSF	AAATCCATGGTTACACTAGTAACGCCTATCATGAATTAGC
EC1.2p-900-NSF	ATAATCCATGGTTATACTAGTGAATAAAAGCATTTGCGTT
35Sen-NcF	ATAGTGGCCATGGAGTCAAAGATTC
35Sen-NhR	ATATATTAGCTAGCATCACATCAATCCACTTGCTT
EC1.2en5-NSF	ATGCAATCCATGGTTATACTAGTGAATAAAAGCATTTGCGTT
EC1.2en5-R	TGGTAAAATTACAGTCTATAACCATCATCT
EC1.2en3-ArF	ACGTTGCCTAGGTGATGGTTATAGACTGTAAT
EC1.2en3-NhR	AATGTATGCTAGCTTAGTGGTGATTTAAGAGTA
EASE-NSF	ATTACATACCATGGTTACACTAGTGCCACGATGCAAATATATCG
EASE-BsR	ACATTTATTGGTCTCAGCCTTAATATCATACGAAAGAG
EASE-BsF	ACATTTATGGTCTCTAGGCCCACGATGCAAATATATCG
EASE-NhR	ATTTATTGCTAGCCTTAATATCATACGAAAGAG
EC1.2en-NHF	CATTCAATCCATGGTAAGCTTGAATAAAAGCATTTGCGTT
CHLI1-IDF	GAAATCTATCTCCTTATCCTGACCACAG
CHLI1-IDR	TTGTTCAGTAGAGTTGATTTCAGTGGCT
CHLI1-seqF	AGAAGCTGAGTAAACTCTAAGCTCACAC
CHLI2-IDF	ATCTATAGAGAGCTCACTCACTAGC
CHLI2-IDR	CCACCTATCTTCGGGTCAATCACAT
CHLI2-seqF	CTTCAATCTTGACCTGCCCTCGTAT
CPC-off-IDF2	CTATGAACTAAAGGCCGTTGGAAGC
CPC-off-IDR	TCATTGACAGCTCGGTAAGTTAGAC
CPC-off-seqF	GTTTCGTGTCTTCAGATTAGTTCGATGT
TRY-off-IDF	AACACAAAATCGCCCTCCATGACTC
TRY-off-IDR	AACTCTTCCTGCTATCAAATCCCAC
TRY-off-seqF	TAAATTAGTTACACCTCGTGCTGAC
5G50230-off-IDF	CACGGATAAGGTTTGTGCTGTGGAT
5G50230-off-IDR	CCGTTTTTATCAGCCGATGCCAATG
5G50230-off-seqF	TACCAGCAACTGCAATGCAATCT
zCas9-IDF3-2	CTGTTCGTCGAGCAGCACAAGCATT
zCas9-IDF5	AATCAGGGAGCAGGCTGAGAATATC
zCas9-IDF6	CGACCCTCATCCACCAGTCGATTAC
rbcS_E9t-IDR	CATTAGAGGCCACGATTTGACACAT
rbcS_E9t-IDR2	TTCCCAATGCCATAATACTCAAACTCAG
lacp-IDF	CCCAGGCTTTACACTTTATGCTTCC

Restriction enzyme sites are underlined.
