# Supplementary material for: Egg cell-specific promoter-controlled CRISPR/Cas9 efficiently generates homozygous mutants for multiple target genes in Arabidopsis in a single generation
Source: Genome Biol. 2015 Jul 21;16(1):144. doi: 10.1186/s13059-015-0715-0 (PMC4507317; doi:10.1186/s13059-015-0715-0)
Supplement: Additional file 3: Figure S4. — Flow chart for the creation of Arabidopsis T1 homozygous mutants via EPC CRISPR/Cas9. Figure S5. Strategy for screening for T1 bi-allelic mutants with no observable phenotypes. Figure S6. The triple mutant can be differentiated from the double mutant. [file 13059_2015_715_MOESM3_ESM.pdf]

Additional file 3: Figure S4

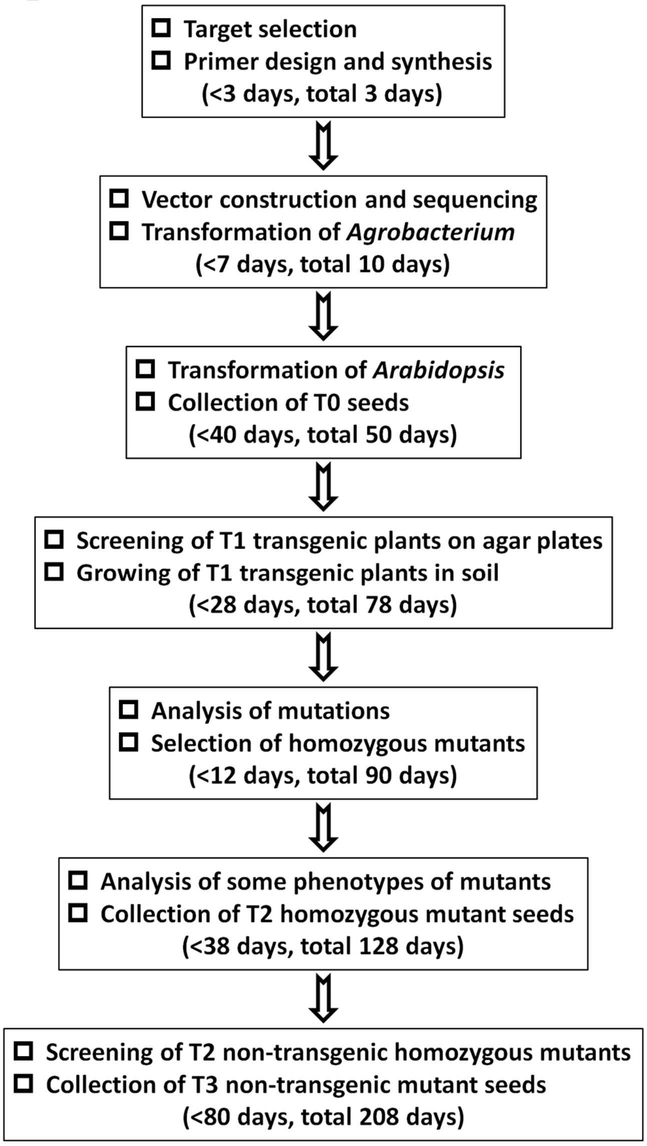

Figure S4. Flow chart for the creation of *Arabidopsis* T1 homozygous mutants via EPC CRISPR/Cas9. The approximate number of days required for each process is indicated.

Additional file 3: Figure S5

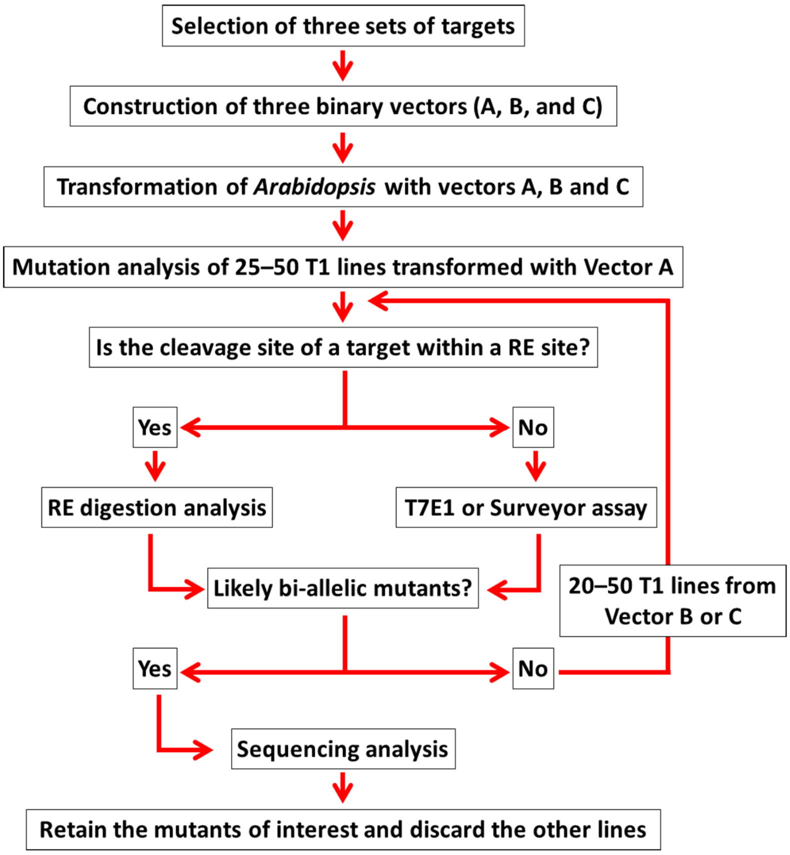

Figure S5. Strategy for screening for T1 bi-allelic mutants with no observable phenotypes. Two out of three sets of targets and vectors B and C are backups to avoid being delayed by possibly difficult targets. RE, restriction enzyme.

Additional file 3: Figure S6

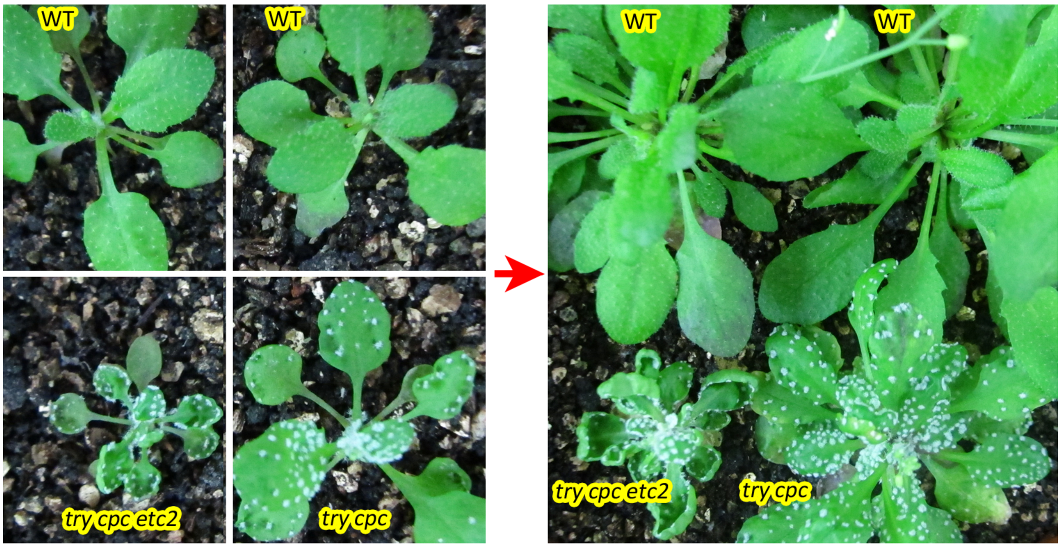

Figure S6. The triple mutant can be differentiated from the double mutant. The triple mutant is a T2 plant derived from #1-T1 (Figure 1) and the double mutant is a previously described T2 plant [1]. The four representative plants were grown in the same pot; two growth stages are shown. The seeds were sown on MS medium, vernalized at 4°C for 3 days, and grown for 7 days. Seedlings were transplanted to soil and allowed to grow for 22 days or 45 days before photographing.
