# Supplementary material for: Egg cell-specific promoter-controlled CRISPR/Cas9 efficiently generates homozygous mutants for multiple target genes in Arabidopsis in a single generation
Source: Genome Biol. 2015 Jul 21;16(1):144. doi: 10.1186/s13059-015-0715-0 (PMC4507317; doi:10.1186/s13059-015-0715-0)
Supplement: Additional file 4: Figure S7. — Thirty-two out of 224 T1 pHEE2B-TRI transgenic plants harbor observable mutations. Figure S8. Fifteen out of 53 T1 pHEE2E-TRI transgenic plants harbor observable mutations. Figure S9. Ten out of 120 T1 pHEE2F-TRI transgenic plants harbor observable mutations. [file 13059_2015_715_MOESM4_ESM.pdf]

Additional file 4: Figure S7

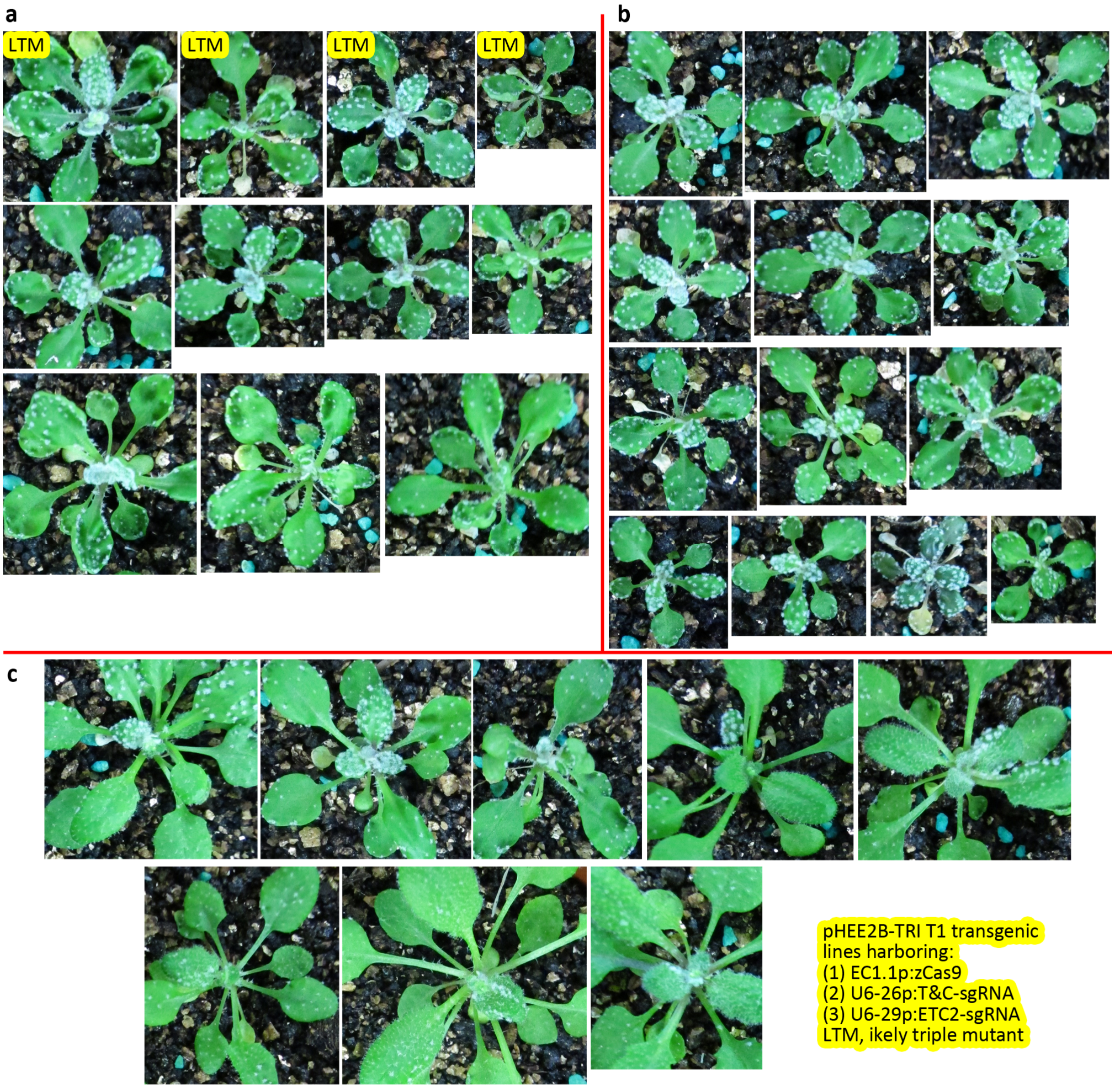

Figure S7. 32 out of 224 T1 pHEE2B-TRI transgenic plants harbor observable mutations. (a) 11 T1 Plants have spoon-shaped leaves (phenotype of triple mutants). Only 4 plants are likely triple mutants (LTMs) whereas the other 7 lines are identified with mosaics in that only parts of leaves of these plants are spoon-shaped. **(b)** 13 T1 plants are likely double mutants or mosaics. **(c)** 8 T1 plants are mosaics.

Additional file 4: Figure S8

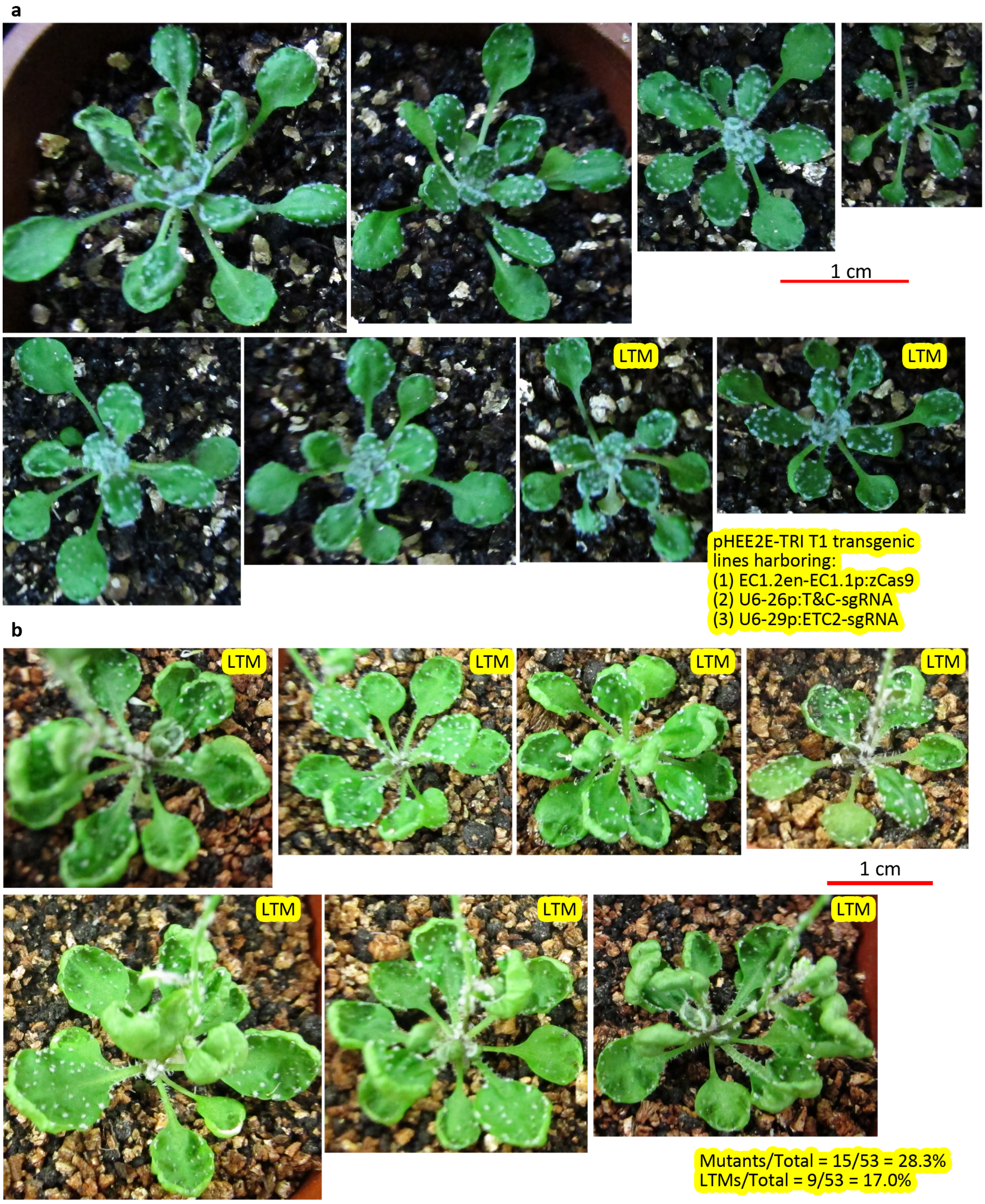

Figure S8. 15 out of 53 T1 pHEE2E-TRI transgenic plants harbor observable mutations. **(a) (b)** Mutants with observable phenotypes from two screenings of T0 seeds, respectively. LTM, likely triple mutant.

Additional file 4: Figure S9

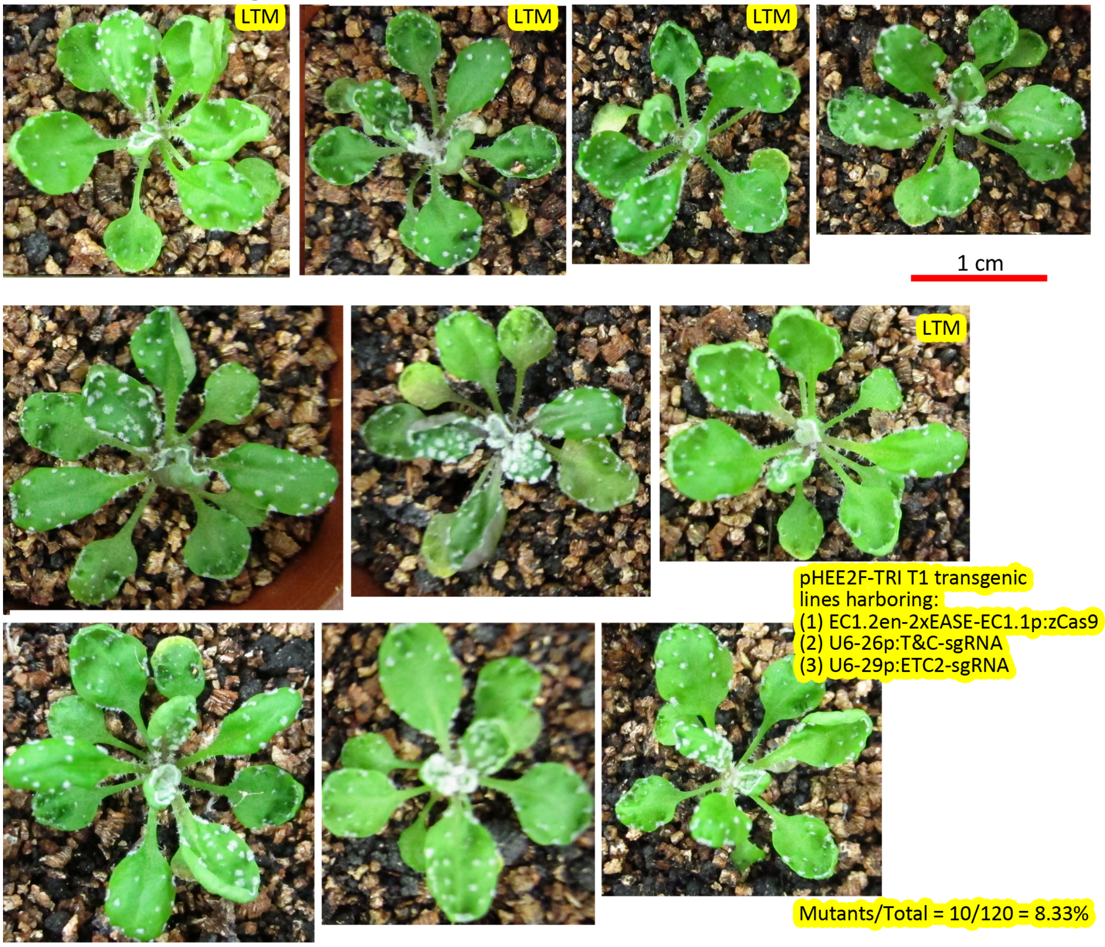

Figure S9. 10 out of 120 T1 pHEE2F-TRI transgenic plants harbor observable mutations. LTM, likely triple mutant.
