# Supplementary material for: Egg cell-specific promoter-controlled CRISPR/Cas9 efficiently generates homozygous mutants for multiple target genes in Arabidopsis in a single generation
Source: Genome Biol. 2015 Jul 21;16(1):144. doi: 10.1186/s13059-015-0715-0 (PMC4507317; doi:10.1186/s13059-015-0715-0)
Supplement: Additional file 5: — Methods S1. Vector construction. [file 13059_2015_715_MOESM5_ESM.pdf]

Additional file 5: Methods S1

Vector construction

All primers used in this study are listed in Additional file 1: Table S3. The vector sequences are provided in Additional file 6. We obtained the U6_26p-SpR-gRNA_Sc-U6_26t cassette by PCR amplification from pHSE401 [1] with primer pair U6-26-HiF2/-XNR. We purified the PCR products, digested them with *Hin*dIII and *Xba*I, and inserted the digested fragment into pHSE401 to replace its *Hin*dIII-*Xba*I fragment, resulting in the production of pH0E401. We obtained the 1010-bp *EC1.2* and 548-bp *EC1.1* promoters including the 5'-UTR by PCR amplification from *Arabidopsis* genomic DNA with primer pairs EC1.2p-NcF/-NhR and EC1.1p-NcF/-XbR, respectively. We purified the PCR fragments, digested them with *Nco*I, and *Nhe*I (for EC1.2p) or *Xba*I (for EC1.1p), and inserted the digested fragments into the *Nco*I and *Xba*I sites of pH0E401, resulting in the production of pHEE401 and pHEE401B. We constructed the vector pHEN401 and pHEN401B in the same manner except that we used pHSN401 in place of pHSE401 [1]. We obtained 35S enhancer (35Sen), *EC1.1* promoter (EC1.1p), and *EC1.2* promoter (EC1.2p, 900-bp) by PCR amplification from pBI121, pHEE401B, and pHEE401 with primer pairs 35Sen-NcF/-NhR, EC1.1p-NSF/-XbR, EC1.2p-900-NSF/-NhR, respectively. We purified the PCR fragments, digested them with *Nhe*I, *Spe*I, and XbaI, respectively. We also generated *Spe*I-digested EC1.2p fragment. We ligated the *Spe*I-digested EC1.1p, or *Spe*I-digested EC1.2p, or *Xba*I-digested EC1.2p, with the *Nhe*I-digested 35Sen and the blunt-end cloning vector pCBC, resulting in the production of pCBC-35Sen-EC1.1p, pCBC-35Sen-EC1.2p-900 and pCBC-35Sen-EC1.2p-565, respectively. We inserted the *Nco*I-*Xba*I fragment of 35Sen-EC1.1p, and *Nco*I-*Nhe*I fragments of 35Sen-EC1.2p-900/565 from the pCBC-derived vectors, into the *Nco*I and *Xba*I sites of pH0E401, resulting in the production of pHEE401D1/2/3. To disrupt the *Xba*I site of *EC1.2* promoter, we obtained the two fragments of *EC1.2* promoter by PCR amplification from pHEE401 with primer pairs EC1.2en5-NSF/-R and EC1.2en3-ArF/-NhR, respectively. We purified the PCR fragments, digested the two fragments with *Xba*I and *Avr*II, respectively. We then ligated the two fragments with pCBC, resulting in pCBC-EC1.2en. We obtained *EC1.1* promoter by PCR amplification from pHEE401B with primer pair EC1.1p-NSF/-XbR. We purified the PCR fragment and ligated it with pCBC, resulting in pCBC-EC1.1p. We inserted the *Nco*I-*Nhe*I fragment of EC1.2 enhancer from pCBC-EC1.2en into the *Nco*I and

*Spe*I sites of pCBC-EC1.1p, resulting in pCBC-2EC. We obtained two 77-bp EASE [2, 3] fragments by PCR amplification from Arabidopsis (Ler) genomic DNA with primer pair EASE-NSF/-BsR and ASE-BsF/-NhR, respectively. We purified the two PCR fragments, digested them with *Nco*I, *Nhe*I and *Bsa*I. We ligated the digested fragments with *Nco*I and *Spe*I-digested pCBC-EC1.1p, resulting in pCBC-3EC. We obtained the EC1.2en fragment by PCR amplification from pCBC-EC1.2en with primer pair EC1.2en-NHF/EC1.2en3-NhR, we purified the PCR products, digested them with *Nco*I and *Nhe*I, and inserted the fragment into the *Nco*I and *Spe*I sites of pCBC-3EC, resulting in pCBC-4EC. We inserted the *Nco*I-*Xba*I fragment of EC1.2en-EC1.1p or EC1.2en-2xEASE-EC1.1p from the pCBC-2EC/4EC, into the *Nco*I and *Xba*I sites of pH0E401, resulting in the production of pHEE401E/F. We constructed the vectors pHEE2A/B/D1/D2/D3/E/F-TRI, pHEN2A/B-TRI, and pHSE2A-TRI, in the same manner as p2gR-TRI-A [1] except that we used pHEE401/401B/D1/D2/D3/E/F, pHEN401/401B, and pHSE401, respectively, in place of pHSN401.We constructed pHEE2A-CHLI in the same manner as pHSE-2gR-CHLI [1] except that we used pHEE401 in place of pHSE401. In short, we obtained a single PCR fragment flanked by two sgRNA targets and two *Bsa*I sites incorporated by primers, we purified the PCR products, and digested them with *Bsa*I and ligated them with one of the *Bsa*I-linearized binary vectors, resulting in the final CRISPR/Cas9 binary vectors. We replaced the *rbcS E9* terminator of pHEE2A-TRI with *Sac*I–*Eco*RI fragment of *nos* terminator from pBI121, resulting in the production of pHEN2C-TRI.

References

1. Xing HL, Dong L, Wang ZP, Zhang HY, Han CY, Liu B et al. A CRISPR/Cas9 toolkit for multiplex genome editing in plants. BMC Plant Biol. 2014;14(1):327.
2. Even-Faitelson L, Samach A, Melamed-Bessudo C, Avivi-Ragolsky N, Levy AA. Localized egg-cell expression of effector proteins for targeted modification of the Arabidopsis genome. Plant J. 2011;68(5):929-37.
3. Yang W, Jefferson RA, Huttner E, Moore JM, Gagliano WB, Grossniklaus U. An egg apparatus-specific enhancer of Arabidopsis, identified by enhancer detection. Plant Physiol. 2005;139(3):1421-32.
